# Robustness and lethality in multilayer biological molecular networks

**DOI:** 10.1038/s41467-020-19841-3

**Published:** 2020-11-27

**Authors:** Xueming Liu, Enrico Maiorino, Arda Halu, Kimberly Glass, Rashmi B. Prasad, Joseph Loscalzo, Jianxi Gao, Amitabh Sharma

**Affiliations:** 1 Key Laboratory of Imaging Processing and Intelligent Control, School of Artificial Intelligence and Automation, Huazhong University of Science and Technology, Wuhan, 430074 China; 2 Channing Division of Network Medicine, Department of Medicine, Brigham and Women’s Hospital, Harvard Medical School, Boston, MA 02115 USA; 3 Genomics Diabetes and Endocrinology, Lund University Diabetes Centre, CRC, Malmö, SE 20502 Sweden; 4 Department of Computer Science, Rensselaer Polytechnic Institute, Troy, NY 12180 USA

**Keywords:** Dynamic networks, Regulatory networks, Robustness, Complex networks, Phase transitions and critical phenomena

## Abstract

Robustness is a prominent feature of most biological systems. Most previous related studies have been focused on homogeneous molecular networks. Here we propose a comprehensive framework for understanding how the interactions between genes, proteins and metabolites contribute to the determinants of robustness in a heterogeneous biological network. We integrate heterogeneous sources of data to construct a multilayer interaction network composed of a gene regulatory layer, a protein–protein interaction layer, and a metabolic layer. We design a simulated perturbation process to characterize the contribution of each gene to the overall system’s robustness, and find that influential genes are enriched in essential and cancer genes. We show that the proposed mechanism predicts a higher vulnerability of the metabolic layer to perturbations applied to genes associated with metabolic diseases. Furthermore, we find that the real network is comparably or more robust than expected in multiple random realizations. Finally, we analytically derive the expected robustness of multilayer biological networks starting from the degree distributions within and between layers. These results provide insights into the non-trivial dynamics occurring in the cell after a genetic perturbation is applied, confirming the importance of including the coupling between different layers of interaction in models of complex biological systems.

## Abstract (web summary)

Robustness is a prominent feature of most biological systems, but most of the current efforts have been focused on studying homogeneous molecular networks. Here the authors propose a comprehensive framework for understanding how the interactions between genes, proteins, and metabolites contribute to the determinants of robustness.

## Introduction

The recent development of high-throughput omics technologies has facilitated the extensive profiling of the different molecular strata comprising living organisms, such as the transcriptome, epigenome, and proteome, providing a more comprehensive picture of the detailed molecular composition of cellular systems. Cellular processes are not only driven by individual molecules, however, but also by the interplay between them. These interactions are conventionally modeled as context-specific molecular interaction networks^1^, such as gene regulatory networks^2,3^, protein–protein interaction (PPI) networks^4^, and metabolic networks^5,6^. Network modeling^7^ has become an effective and widely used approach in the analysis of cellular systems. While the study of the static topology of these networks has been successful in various applications, such as disease gene prioritization^8^, disease biomarker discovery^9^, and disease diagnosis and subtyping^10^, substantial insights can be gained by analyzing the properties of dynamical processes evolving over the nodes and edges of the network. These processes are usually defined to simulate the effects of environmental changes, internal perturbations, the onset of diseases, or random failures occurring in the network^11^.

An established approach to quantifying the effect of perturbations in a biological system is the analysis of the system’s robustness, defined as its ability to maintain stable functioning despite various perturbations^12,13^. In biological systems across all scales, from cells to organisms, robustness is attained by a combination of five mechanisms: feedback control, structural stability, redundancy, modularity, and adaptation^14^. For example, by applying percolation theory to the analysis of the robustness of biological networks^15^, Jeong et al.^16^ found strong connections between the centrality of a protein and its lethality. In other work, metabolic networks have been shown to be exceptionally robust^17^, hinting as to why organisms can survive in a wide range of environmental conditions. The analysis of the robustness of molecular networks under perturbations has become an efficient tool for uncovering disease mechanisms at the molecular level^18^. Most of these studies focus on the investigation of single molecular networks. Molecular networks are not independent, however, and processes can span multiple molecular layers simultaneously, generating intricate patterns that are difficult to uncover when networks are analyzed separately^11^. For example, in a cell, genes can activate or inhibit other genes, and this regulation is operationalized through physical protein–protein and protein–DNA binding. Proteins can, in turn, affect metabolic reactions through enzyme catalysis. In these cases, exploring networks of molecules of the same kind in isolation can ultimately lead to an incomplete or even incorrect picture of the problem. Thus, accounting for the interactions between different molecular networks is critical for understanding cell dynamics and functionality.

In network science, systems composed of multiple interacting networks^19–22^ have attracted considerable attention owing to the discovery of novel structural and dynamical features in coupled cases that differ from those observed in uncoupled cases. In the past decade, the mathematical frameworks for characterizing the robustness of a network of networks^20^ or multilayer networks^22^ have been studied in various settings, such as full interdependency^19^, partial interdependency^23^, interconnections^24^, spatially embedded networks^25^, multiple supports^26^, directed networks^21^, multiple networks^27^, and many more^28–30^. The robustness of multilayer networks has a broad impact on infrastructure networks^31^, ecological systems^32^, social networks^33^, and financial networks^34^. Recently, the growing availability of massive genomic, proteomic, and metabolomics data has stimulated the construction of multilayer biological molecular networks^35–37^. For example, Shinde et al.^38^ proposed a multiplex network composed of six different PPI layers representing different life stages of *Caenorhabditis elegans*, showing varying degree–degree correlation and spectral properties across the nematode’s life cycle. Bennett et al.^39^ found functional communities across layers in a two-layer PPI network of yeast, where one layer is connected by physical interactions and the other by genetic interactions. In this context, different layers model different kinds of interactions. Klosik et al.^40^ designed a vast, directed, biological molecular network, called the interdependent network of gene regulation and metabolism, which is composed of three types of biological molecules: genes, proteins, and metabolites. For multicellular organisms such as humans, Didier et al.^41^ and Valdeolivas et al.^42^ investigated the community structure in multiplex biological molecular networks, which is composed of three or four biological networks sharing the same set of genes/proteins, with the nodes in each layer connected by different types of interactions, such as co-expression or physical interactions.

Despite these advances, our understanding of determinants of the robustness and lethality of biological systems is limited^43,44^. The difficulty is rooted in three independent factors, each with its own complexity: (1) A comprehensive framework integrating heterogeneous sources of data of human molecular networks is still lacking. The integration of various molecular data, such as gene regulatory, PPI, and metabolic networks, is a challenging problem because these components have completely different features and modes of interaction within an organism, which are measured in fundamentally different ways. (2) We lack general models as to how gene perturbations propagate and affect the downstream functions of the cell and its components. It is difficult to capture a holistic picture of the process by which a specific genetic perturbation propagates across a biological network. However, modeling the effect of gene perturbations with reasonable mechanisms could give us a better understanding of the complex dynamics of the cell’s molecular machinery. (3) Most previous theoretical frameworks are agnostic to their applied setting and deal with networks of the same type, which are either all undirected^20^ or all directed^21,45^, and in which the interdependence relations are random. By contrast, biological networks include interactions that can be both directed and undirected and of different types, and the topological structure of the interlayer links follows specific wiring patterns that are far from random. In addition, different interaction networks can vary considerably in size and connectivity. Thus, developing a general framework by which to analyze the robustness of multilayer biological networks remains an unsolved problem in interdependent networks^46^.

## Results

### Construction of the multilayer biological network

**Fig. 1:**
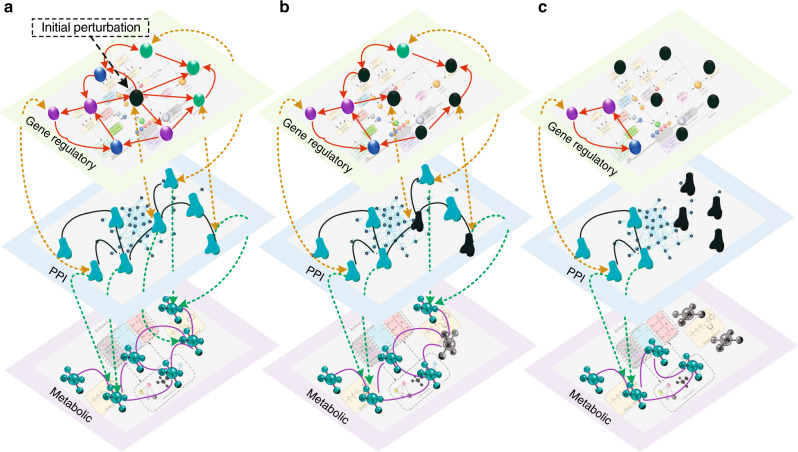
Schematic demonstration of the cascading failure process in multilayer biological molecular networks. The multilayer model includes a gene regulatory network in which the genes (ellipses) are linked by regulatory relations (red directed links), a PPI network in which proteins (bone shapes) are linked by physical interactions (black undirected links), and a metabolic network in which metabolites (molecule shapes) are connected by chemical–chemical interactions (purple undirected links). The gene regulatory and PPI networks are connected by bidirectional interdependency links (yellow dashed lines). From the PPI to metabolic networks, there are multiple supporting links (green dashed lines). **a** Initially perturb a gene in the gene regulatory network causing such gene to stop functioning (represented by a black ellipse). **b** The target genes of the perturbed genes fail (black ellipses), and their corresponding proteins stop functioning, represented by black bone shapes. **c** The proteins that disconnected from the largest connected component fail (black bone shapes), and the metabolites losing all supports from the PPI network stop functioning (black molecule shapes).

According to the central dogma of molecular biology, DNA is transcribed into RNA which is then translated into protein products. Moreover, many proteins can regulate metabolic reactions through enzyme catalysis. Based on these well-known relationships, we constructed a multilayer network by aggregating three major biochemical networks that govern cell function: a gene regulatory, a PPI, and a metabolic layer, as shown in Fig. 1.

We first construct three layers of biological molecular networks (see Methods section for details):

1. Gene regulatory network. We use two types of gene regulatory networks in our work: a general gene regulatory network and three tissue-specific gene regulatory networks^18^. The general gene regulatory network is generated by curating the binding motifs of a subset of 695 unique human transcription factors, and the tissue-specific gene regulatory networks are curated from the FANTOM5 database^47^.
2. PPI network. We use the comprehensive human interactome built by Cheng et al.^48^, which integrated multiple databases with experimental evidence. After removing the self-loops, we obtain a PPI network whose largest connected component consists of 15,906 proteins connected by 213,874 links.
3. Metabolic network. The metabolic network is constructed by curating the biochemical–biochemical (metabolite–metabolite) interactions from the STITCH database^49^ and then mapping to metabolites in the Human Metabolome Database (HMDB)^50^. This metabolic network had statistically significant enrichment of overlapping edges with analogous metabolic networks constructed using genome-scale metabolic models such as Recon^51^ (Table S2). For a detailed comparison between STITCH- and Recon-based metabolic layers, see Supplementary Note 5.
4. Connections between Gene regulatory and PPI networks. We connect the protein-coding genes in the gene regulatory network directly to their protein products in the PPI network. These connections result in 10,255 bidirectional interlayer links between the general gene regulatory network and the PPI network.
5. Links from PPI to metabolites. Protein–biochemical links are compiled from the STITCH database^52^ and are directed from the protein to the metabolic layer, as we make the simplifying assumption that the perturbation of an enzyme affects the metabolic reactions it regulates, and the cases where enzyme levels may, in turn, be affected due to feedback loops are neglected. Note that proteins and metabolites are connected in a many-to-many relation, since multiple enzymes and chemicals can participate in the same reaction, and multiple proteins can be associated with multiple metabolites. The interconnections between the PPI network and the metabolite network are obtained through the biochemical-protein links in the STITCH database. For the 15,906 proteins and the 1269 metabolites in the multilayer network, we have 141,283 directed interlayer links connecting 12,039 proteins to 1211 metabolites.

To model the functionality and robustness of multilayer biological networks, we define a cascading failure mechanism simulating the effect of a perturbation in the network. From the molecular viewpoint, the cascade corresponds to a process whereby a number of perturbed transcription factors lose their ability to regulate their targets, resulting in some genes being left unregulated in the regulatory network, ultimately affecting the expression of the proteins for which they code in the PPI network. The altered expression of these proteins, in turn, disrupts the metabolic reactions they regulate.

The process is summarized below and shown in Fig. 1. Each node of these networks is assigned a two-state variable, either functional or dysfunctional, and all the nodes are initially set as functional nodes. When a node becomes dysfunctional, it is removed from the network. The perturbation originates from a set of predefined target genes (TGs) in the gene regulatory network, simulating a loss of functionality due to, for example, mutations or gene knockouts. Since the TGs lose their ability to regulate other genes, both the TGs and the genes they regulate in the gene regulatory network become dysfunctional. As a consequence, the corresponding protein products of all of the involved genes become dysfunctional. After the removal of these proteins, the functional proteins that are left disconnected from the largest connected component of the PPI network become dysfunctional, following the assumption that any protein must be connected to the larger network of interacting proteins for it to be a part of the functioning cellular machinery^53–55^, along with their corresponding protein-coding genes. The regulated genes of the newly dysfunctional genes are then removed and the process continues in a cascading fashion until a stable state is reached.

In a metabolic network, each metabolite has multiple support links from the protein–protein interaction network. A metabolite stops functioning if a fraction *f*_P2M_ of its supporting proteins becomes dysfunctional, where *f*_P2M_ is a constant between 0 and 1. As an additional modeling choice, a metabolite is functional at any given time only if it belongs to the largest connected component. As shown in Fig. 1 and Supplementary Note 1, the perturbation in the gene regulatory network can cause cascading failures to propagate across the gene regulatory and PPI networks. When the process comes to a halt, the remaining nodes are identified as the final functional component.

### Influential genes are enriched in essential and cancer genes

To investigate how the couplings between the gene regulatory and PPI networks contribute to defining the system structure and function, we compared the effects of the above-mentioned perturbation process, hereby referred to as a coupled process, to the outcomes of a perturbation only affecting the PPI network alone, or an uncoupled process, which removes the perturbed protein from the isolated PPI network directly. In order to provide a fair comparison between the two processes, we only consider perturbations of genes that are associated with their corresponding protein products, while genes that have no connections to the PPI layer are excluded from the analysis. In the PPI network of both uncoupled and coupled cases, we characterize the contribution of each node to the system’s integrity by measuring the final functional network size, ${f}_{{\rm{S}}}^{{\rm{P}}}$, when that node is removed. A smaller final fraction of functional nodes indicates a larger contribution of the target node to system integrity. Thus, we assign an influence score ($1-{f}_{{\rm{S}}}^{{\rm{P}}}$) to each node to evaluate its influence on the system’s robustness, where ${f}_{{\rm{S}}}^{{\rm{P}}}$ is the final functional size of the PPI network.

**Fig. 2:**
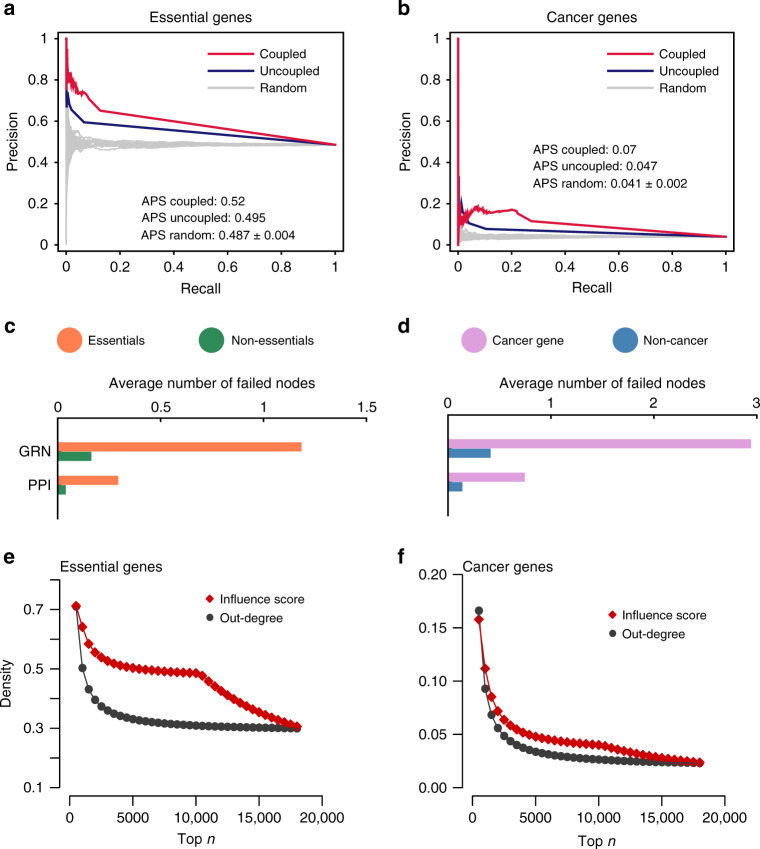
Comparison between the coupled and uncoupled cases. Average precision–recall (PR) curves of the coupled (red) and uncoupled (blue) influence scores in the prioritization of essential (**a**) and cancer (**b**) genes. The gray PR curves represent 100 random node rankings. On the right of each plot are listed the average precision scores (APS) of the three ranking strategies evaluated from the corresponding PR curves. The performance of coupled influence scores in prioritizing essential and cancer genes are, respectively, 5.05% and 48.94% higher than that of uncoupled influence scores. In the coupled case, the removal of a single-gene not only causes one-time failures as those in the uncoupled cases but also causes a second or third round of cascading failures. The average numbers of nodes failing in the second round caused by the removal of essential (**c**) and cancer genes (**d**) are, respectively, higher than that of removing the nonessential and non-cancer genes, explaining why the coupled case performs better in prioritizing essential and cancer genes. In addition, the densities of **e** essential and **f** cancer genes among the top *n* genes ranked by influence scores (red diamonds) are higher than that ranked by out-degrees (black circles). For the genes of the same influence scores or of the same out-degrees, we randomly put their orders 100 times and compute the average densities. It indicates that the influence scores perform better than out-degrees in uncovering the connections between network topology and biological mechanisms.

To select a ground truth, we compiled two sets of genes that are recognized to have important biological roles. The first set consists of the biologically essential genes, i.e., genes that are indispensable for supporting cellular viability, collected from the database of essential genes^56^. The second set is composed of the genes that have been causally implicated in cancer development, integrated from the Cancer Gene Census^57^. We note that, while a number of network-based approaches have been developed for the in silico prediction of essential genes^58^, including those that rely on genome-scale metabolic models^59^, here we employed essential gene information from the literature as a general criterion to assess the biological importance of the prioritized genes. We calculated the precision–recall curves of coupled and uncoupled influence scores in recovering the sets of essential and cancer genes (see Supplementary Note 4). As shown in Fig. 2a, b, the coupled influence scores yield higher precision–recall scores compared to the uncoupled and random scores, denoting the higher descriptive power provided by the layer couplings. In the coupled case, the removal of a single-gene does not only cause one-time failures like those in the uncoupled cases, but also causes the second or even third round of cascading failures. We find that the average number of genes/proteins that became dysfunctional in the second round as a consequence of the removal of an essential or cancer gene is higher than that of removing a nonessential or non-cancer gene, as shown in Fig. 2c, d, explaining why the coupled case performs better in prioritizing essential and cancer genes than the uncoupled case. To test the generalizability of this result, we sequentially replaced the gene regulatory layer with three tissue-specific gene regulatory networks, namely, forebrain, lymphocytes, and lung (see “Methods” section). In all three cases (see Fig. S6), the coupled influence scores are more informative than the uncoupled scores in detecting essential and cancer genes.

The same result holds when using different criteria for selecting essential genes: (1) probability of haploinsufficiency (Phi), (2) probability of loss-of-function intolerance (pLI), and (3) essential genes found by Dickinson et al.^60^. Genes with high scores of essentiality, as measured by these metrics, are associated with higher influence scores, and they are more prevalent among the genes with high influence scores in the coupled model compared with those of the uncoupled model (Fig. S7). Note that the influence score in the coupled case incorporates the contribution of a gene to the integrity of the PPI network. We further test the performance of coupled influence scores in prioritizing disease genes categorized by their association with Mendelian or complex diseases. We divide the disease genes into MC (both Mendelian and Complex), MNC (Mendelian but Not Complex), and CNM (Complex but Not Mendelian) disease genes, as defined in ref. ^61^. The influence scores of genes show higher performance in prioritizing CNM disease genes (Fig. S8), suggesting that complex-disease genes have more cohesive connections to their surroundings than Mendelian disease genes. As an additional confirmation of this result, we assessed the significance of the overlap between the top influential genes and the disease-related gene sets (MC, MNC, and CNM) in two different ways. First, by computing the p-value of a hypergeometric test of overlap between the two sets, and second, by comparing it to a null distribution of overlaps with random gene sets of the same size and degree distribution (see “Methods” section). We observe in both cases (see Figs. S9 and S10) that influential genes are enriched in complex-disease-related genes, while this enrichment is nonsignificant or marginally significant in the case of Mendelian disease genes. This observation aligns with the current understanding of complex diseases, which are hypothesized to stem from the interactions among a multitude of genes, requiring a higher degree of influence on their surroundings. By contrast, since Mendelian genes are, by definition, the primary cause of the disease phenotype they induce, their influence scores are indistinguishable from random chance.

Since the proposed failure mechanism depends on the out-degrees of the perturbed genes, we investigated to what extent the information provided by the influence score is different from the simple out-degree measure. We compute the fraction of essential, disease, and cancer genes among the sets of top *n* genes ranked by the influence scores and out-degrees, repeating the operation for each *n*. To account for the ambiguity in ranking caused by genes with the same out-degree or influence score, we randomly shuffled the ranks of the groups of genes corresponding to the same values 100 times and computed the average ratios. As shown in Fig. 2e, f, genes ranked by the influence score are enriched in larger fractions of essential and cancer genes compared to genes ranked by their out-degree, indicating that influence scores have a higher sensitivity in discerning the genes involved in critical cellular processes.

Finally, to demonstrate the added benefit of considering a multilayer structure and to compare our approach with valid alternative methods that identify biomolecular entities with impact on the robustness of biological systems, we used an established in silico single-gene deletion approach based on flux balance analysis (FBA)^51^ on the metabolic layer only (see Supplementary Note 5). The essential genes and reactions found using this approach were limited in number (Figs. S21 and S22), supporting the notion that the influence score provides a complementary and extended view to essentiality that is more focused on robustness, identifying many additional genes that are potentially crucial to the functioning of the system as a whole.

### Perturbation of metabolic disease genes and metabolic network dysfunction

Gene perturbations can propagate to the metabolic network through the failure of enzymes. For example, consider the gene TCF7L2, one of the most replicated type 2 diabetes mellitus (T2D) susceptibility genes^62^. Its perturbation generates a cascade of failures that leads to the removal of o-hydroxyphenylacetic acid and lipid peroxidation in the metabolic layer. Lipid peroxidation has been observed to be directly associated with T2D^63^, while o-hydroxyphenylacetic acid is formed from phenylalanine, an amino acid that is consistently associated with T2D risk^64^. As a more extensive experiment, we assessed the consequences of perturbing a group of metabolic disease genes in the gene regulatory network on the integrity of the coupled metabolic network. We integrated multiple sources from large-scale genome-wide association studies (GWAS) published in the literature and data from the GWAS Catalog^65^ to compile three sets of genes that are associated with dyslipidemia, hypertension, and type 2 diabetes (see “Methods” section). From each set of metabolic disease genes, we randomly perturb a fraction *p* of genes and calculate the final functional metabolic network size, for *p* ∈ {0.1, 0.2, . . . , 1}. In order to control for node degree, for each metabolic disease gene set we generate a population of random gene sets of the same size and similar degree distribution as the original set and repeat the perturbation process described above.

**Fig. 3:**
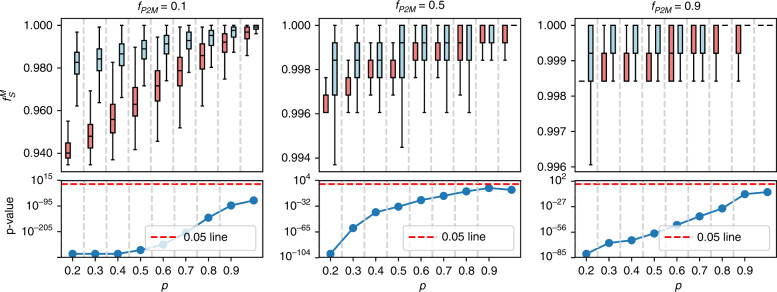
Targeting dyslipidemia-related genes (red boxes) causes more damage to the metabolic network than degree-preserving random attacks (blue boxes). (Top) The fraction of functional nodes after perturbations for several values of the remaining fraction *ps* of metabolic disease genes and threshold proportion *f*_P2M_ (a metabolite fails if more than *f*_P2M_ fraction of supporting proteins fail); (bottom) *p* values of the one-sided Mann–Whitney test between the distributions of the functional node-set sizes in the targeted and random case. Lower values indicate a higher degree of damage to the network. The result of each bar is calculated based on 1000 random realizations. For each distribution, boxes indicate the quartiles, whiskers extend to an additional 1.5 * IQR interval, and the medians are represented by a black line.

As shown in Fig. 3 and Figs. S11–S16, perturbations targeting these gene sets cause more damage to the metabolic network than random perturbations. For example, Fig. 3 shows the comparison between targeted perturbations (red boxes) of the dyslipidemia-related genes and random perturbations (blue boxes). In addition, we find that this result holds regardless of the value of the threshold *f*_P2M_ in the failure mechanism of the metabolic network (if more than a fraction *f*_P2M_ of supporting proteins fail, then the metabolite fails).

### Robustness of the multilayer biological network

To define a baseline for the robustness of the real biological molecular networks, we considered multiple versions of randomized models: (1) intra-layer randomized versions, where the randomization occurs within the gene regulatory, PPI, or metabolic layers; and (2) interlayer randomized versions, where the gene–protein or the protein–metabolite connections are randomly rewired. For each randomization scheme, we defined three modes of rewiring: neutral, assortative, and disassortative rewiring. The randomized versions of the PPI and metabolic networks are generated by multiple rewiring of pairs of edges. At each step, we randomly select two pairs of connected nodes, such as (A, B) and (C, D), then (1) for neutral randomizations, connect node A with node C or D, then connect the remaining two nodes; (2) for assortative randomizations, connect the node with the highest degree among these four nodes to the node of the next highest degree in this subset and connect the remaining two nodes; (3) for disassortative randomizations, connect the node with the highest degree among these four nodes to the node of the lowest degree in this subset and then connect the remaining two. During these processes, multiple links and self-loops are forbidden.

In addition, for the directed gene regulatory network, we assessed how the in-degree and out-degree correlation of the same node affects the robustness of the model. We randomize by keeping both the in-degree and out-degree distributions unchanged, and rewire the network so that nodes with higher out-degrees tend to have higher in-degrees in the assortative versions. Assortative randomization in the directed gene regulatory network is realized by maintaining the in-degree and out-degree distributions but increasing the correlations between the in-degree and out-degree of each node^66^ (see Fig. S5). Assortative randomization in couplings involves connecting the high-degree nodes in one layer to the high-degree nodes in the other layer. Similarly, disassortative randomization is realized by decreasing the in-degree and out-degree correlations or connecting the high-degree nodes to the low-degree nodes.

**Fig. 4:**
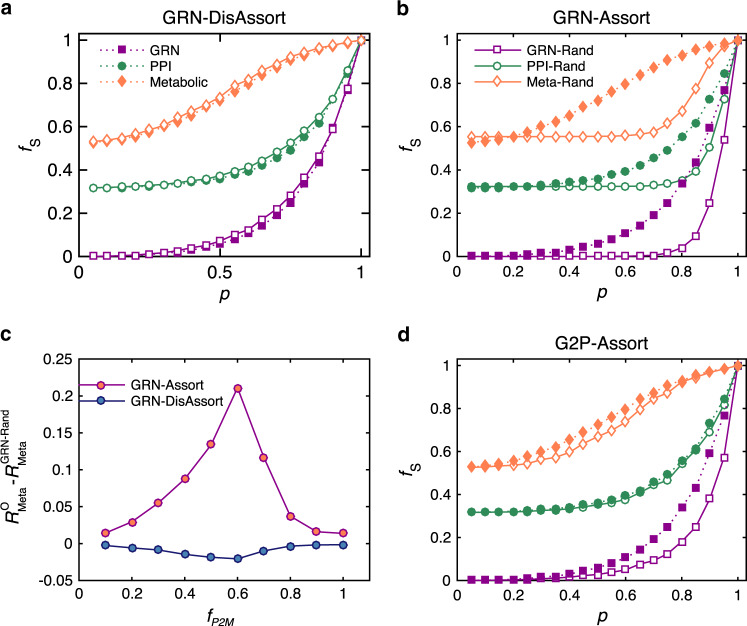
Comparing the robustness of the real biological multilayer networks (filled symbols) and randomized models (unfilled symbols). The results are averaged over 30 realizations. Higher values mean greater robustness of the system. The real model has comparable robustness as **a** the model that is disassortatively randomized in the gene regulatory network, and higher robustness than **b** the model that is assortatively randomized in the gene regulatory network. Here, the result in the metabolic layer is evaluated by setting the threshold *f*_P2M_ = 0.7, and the results under other threshold values are shown in (**c**), where ${R}_{{\rm{Meta}}}^{{\rm{O}}}$ and ${R}_{{\rm{Meta}}}^{{\rm{CRN-Rand}}}$, respectively, represent the integral size of the functional metabolic network in the real and randomized models, with *p* varying from 0 to 1. If the value ${R}_{{\rm{Meta}}}^{{\rm{O}}}-{R}_{{\rm{Meta}}}^{{\rm{CRN-Rand}}}$ is larger than zero under different thresholds, the real model is more robust than the assortatively randomized model (GRN-Assort). The real metabolic layer has comparable robustness with the disassortatively randomized model (GRN-DisAssort), since ${R}_{{\rm{Meta}}}^{{\rm{O}}}-{R}_{{\rm{Meta}}}^{{\rm{CRN-Rand}}}$ is near zero under different thresholds; **d** the real model is also more robust than the randomized model in which the gene–protein connections are assortatively rewired.

The robustness of the multilayer biological network can be evaluated by the final functional sizes in the three layers: ${f}_{{\rm{S}}}^{{\rm{G}}}$, ${f}_{{\rm{S}}}^{{\rm{P}}}$, and ${f}_{{\rm{S}}}^{{\rm{M}}}$, where *f*_S_ is a general metric representing any layer after randomly removing 1 − *p* fraction of genes. The higher value of the final functional size indicates higher robustness. Alternatively, robustness can be evaluated by the integral size of the functional network size ($R=\mathop{\int}\nolimits_{0}^{1}{f}_{{\rm{S}}}\ {\rm{d}}p$), with *p* varying from 0 to 1. We find that the robustness of the multilayer molecular network is comparable to or higher than the robustness of the randomized models. For the disassortatively and neutrally randomized models, their robustness is comparable to the real models, as shown in Fig. 4a and Figs. S13–S16. We find that the real model is more robust than the following two randomized models: (1) randomization in the gene regulatory network maintaining the in-degree and out-degree distributions and increasing the in-degree and out-degree correlations (Fig. 4b, c); (2) randomization of the couplings between the gene regulatory and PPI networks maintaining the degree distributions in gene regulatory and PPI networks but increasing the degree correlations between the connected gene–protein pairs (Fig. 4d).

### Comparison of numerical and analytical solutions

In a typical scenario, to quantify the multilayer network’s robustness, one needs to perform simulations on large-scale networks, which is time-consuming and requires significant storage capacity. In particular, the biomolecular networks to which our approach is applicable can vary greatly in terms of size and density. Therefore, we derived an analytical formulation to estimate the functional network sizes in a computationally efficient way.

We first derive equations for calculating the functional network size in the gene regulatory network after initial perturbations on the 1 − *p* fraction of genes (see “Methods” section). The functional network size in the PPI layer after the perturbation process converges can be calculated through the generating function formalism and percolation theory^19^. Most previous frameworks of robustness in multilayer networks are proposed under the assumption that the connections between layers are random, i.e., that the degrees of two connected nodes in two layers either have no correlations^23^ or follow specific patterns^36^, which usually do not hold in real cases. In multilayer biological networks, the analytical estimation of the number of failures propagating from the gene regulatory layer to the PPI layer is challenging since the connections between the two layers are not purely random. We propose an analytical method to determine two equivalent coupling strengths^23^, *q*_G_, and *q*_P_, to quantify, respectively, the fraction of genes that depend on proteins of the PPI network, and the fraction of proteins that depend on genes of the gene regulatory network (see Supplementary Note 2). By using these two coupling strengths, the connections between the gene regulatory and PPI networks can be treated as random in the theoretical calculation.

Next, we present the solution for the final functional network sizes step-by-step according to the cascading process between the gene regulatory network and the PPI network. At the final stage of the cascading process, the final fractions of functional nodes in the gene regulatory and PPI networks are, respectively, *ψ*_m_ and *ϕ*_m_ (see Eq. (5) in the “Methods” section). In the metabolic network, a metabolite node fails if a fraction of more than *f*_P2M_ of its supporting proteins fails. By applying percolation theory, the final functional node size of the metabolic network is ${f}_{{\rm{S}}}^{{\rm{M}}}$ (see Eq. (6) in the “Methods” section).

**Fig. 5:**
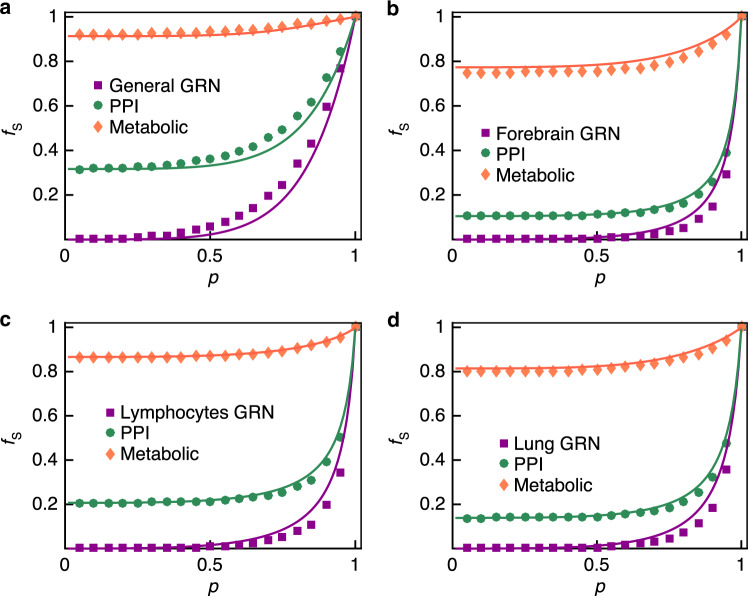
Theoretical predictions on the robustness of the multiple molecular networks. The final functional node sizes in the multilayer molecular networks after randomly removing 1 − *p* fraction of genes from the general gene regulatory network (**a**), and from the tissue-specific networks of the forebrain (**b**), lymphocytes (**c**), and lung (**d**). The solid lines represent the theoretical predictions and the markers represent the simulation results obtained by averaging over 30 realizations. Note that the PPI and metabolic layers considered in these four panels are the same; the value of *f*_P2M_ is set to 1, i.e., a metabolite fails when all of its supports fail. The correlation coefficient between the theory and simulation results is around 0.9918 (see Table S1), indicating that the theoretical results (solid lines) match the simulation results (symbols).

To verify the proposed framework, we first apply it to a synthetic model composed of three layers of Erdős–Rényi (ER) networks. We find that our framework accurately predicts the final functional network size in multilayer ER networks (see Supplementary Note 3). Next, we repeat the calculations for the three-layer biological network described above. We evaluate the fractions of functional nodes at each stage of the perturbation, finding that the fractions of functional nodes in each cascading stage agree with the numerical simulations, as shown in Fig. 5a. To test the generalizability of our framework on multilayer biological networks with arbitrary degree distributions, we sequentially replace the gene regulatory layer with three tissue-specific gene regulatory networks, namely forebrain (Fig. 5b), lymphocytes (Fig. 5c), and lung (Fig. 5d). We find that in all three cases, our analytical framework correctly predicts the final sizes of functional nodes in these multilayer molecular networks.

### Sensitivity analysis and comparative studies

To assess the generality of our results, we evaluated their stability with respect to a change of modeling assumptions and to alterations in data due to noise. We considered multiple experiments in which we varied several aspects of our framework to observe their influence on the results. As a consistent benchmark, in each case, we measured the enrichment of the modified influence score in essential and cancer genes.

We tested the dependence of the influence measure on the quality of the network data we are using since it is well-known that biological interaction networks are affected by considerable noise. We simulated a certain degree of noise in the data by randomly adding/removing a fraction of links from the gene regulatory network and the PPI network. We tested the effect of the random addition/removal of 1% of links and then repeated the same test for an increasing fraction of 2%, 3%, ..., 20%. We then calculated the average precision score (APS) of the coupled and uncoupled case in prioritizing essential and cancer genes.

As shown in Figs. S23 and S24, the performance in the detection of essential and cancer genes is only mildly affected or unaffected by the addition or removal of edges in both the gene regulatory or PPI network. This finding suggests that the influence measure depends on specific structural patterns of the network that are resistant to noise in the data.

We also contemplated a possible modification as to how the perturbation process evolves. Our original model is defined such that once a gene in the gene regulatory layer is perturbed, then all of its out-neighbors lose their functionality. We parameterized this assumption such that, if a gene fails, then only a certain fraction *f* of its out-neighbors also fail. This increase in the complexity of the model simulates those cases in which genes that are the target of a perturbed TF still remain functional because of redundant pathways/regulation by other TFs. We tested this modified rule under the settings *f* = {0.1, 0.2, . . . , 1}. As shown in Figs. S25 and S26, we observe that the performance in prioritizing essential and cancer genes (measured by the APS) is quite stable across all the considered values of *f*.

Next, we verified that the improvement in the performance of the coupled case over the uncoupled case is due to the informativeness of interlayer couplings and not simply a result of the increase of information given by the wider range of network interactions affecting the perturbation process in the coupled case (i.e., gene regulatory and protein–protein interactions as opposed to PPI in the uncoupled case). We reduced the coupling strength between the gene regulatory network and assessed the performance in the coupled case with 0%, 10%, 20%, ..., 100% interdependence links, where the 0% case represents the uncoupled case and the 100% case represents the original coupled case. We find that the APS increases as the coupling strength increases, indicating that interlayer information is crucial for detecting important genes in the coupled system (see Figs. S27 and S28).

As additional confirmation of this result, we generated a merged network that consists of the union of the edges of the GRN and the PPI. We then executed the uncoupled perturbation, as defined previously, on the merged network. Our reasoning was that the uncoupled measure still has access to the same amount of intra-layer information as the coupled one, except for the interlayer edge information, which represents the coupling between the two systems. As we show in Fig. S29, in the prioritization of both essential and cancer genes the uncoupled measure on the merged network (merged in the figure) has a performance that is higher than the uncoupled measure, but still lower than the coupled one. This finding suggests that there is a net gain in considering how the two layers are coupled, in addition to their raw static information content.

## Discussion

To uncover how the couplings between molecular networks influence their biological functions, we propose a minimalist model of multilayer molecular networks encompassing regulatory, protein–protein, and metabolic interactions, and develop a theoretical framework for analyzing the system’s robustness. We define a perturbation process that roughly simulates the cascade of effects occurring in the network when a group of genes is perturbed. We show that our analytical formulation correctly predicts the number of functional nodes at each stage of the cascading process. In this framework, we find that the topology of the proposed multilayer network is more robust than that of randomized models. This finding suggests that molecular networks may have evolved to avoid developing strong degree–degree correlations, so as to increase the system’s robustness under perturbation.

We define an influence score characterizing the contribution of each gene to the system’s robustness, and find that essential and cancer genes are enriched in higher scores compared to random chance. In addition, to assess the contribution of the connections between different molecular layers, we compare the results above with the effects obtained by a perturbation process acting only on the isolated PPI network, finding that the multilayer system achieves superior performance in prioritizing essential and disease genes. Similarly, whereas FBA-based approaches measure the impact of genes directly encoding reactions within the single metabolic layer, our dynamical multilayer approach measures the effect of gene perturbations cascading down the gene regulatory and protein–protein interaction networks to the metabolic network layer. Furthermore, targeting a group of metabolic disease genes causes significantly more damage in the metabolic network when compared to random perturbations, denoting the non-trivial association between these genes and the metabolic processes they regulate.

The results above are complementary. On the one hand, we find that the coupled perturbation process accurately predicts genes that are important for biological processes and survival; on the other hand, we find that perturbing biologically important genes, defined a priori, causes more damage to the overall system’s integrity than perturbing other randomly chosen genes. As shown by these results, the analysis framework proposed in this work allows the integration of heterogeneous sources of data to study the robustness of human molecular networks, opening new avenues of investigation on their organizing principles and dynamics. Future directions of this work are twofold: at a theoretical level, an important unmodeled factor in the analytical formulation is the correlation between in-degree and out-degree of the gene regulatory layer; at the biological level, there are additional molecular mechanisms that have major roles in determining the robustness of a cellular system, such as gene methylation, noncoding RNA regulation, and post-translational modification of the proteome. Importantly, increasing evidence suggests the existence of feedback mechanisms such as the metabolic control of gene expression, whereby specialized transcription factors are activated in response to changes in metabolite levels or metabolic enzymes function as transcriptional coregulators^67^. Future studies that aim to extend the current work could incorporate such mechanisms for an even more realistic coupling of the biological layers.

One additional avenue of investigation for improving this model is the inclusion of detailed molecular interaction parameters within the network, such as protein binding affinities, allowing for a generalization of the methodology to weighted networks. In a similar vein, while we have opted for a metabolic layer that will maximize metabolite coverage and connectivity to the other layers, we note the use of flux balance analysis-capable genome-scale metabolic models such as Recon as a potential future extension of our work. Indeed, the significant overlap of edges between STITCH- and Recon-based metabolic networks suggests the concordance of the metabolic layers built using the two methods and supports the utilization of stoichiometric and constraint-based techniques that allow for a dynamical depiction of the human metabolic machinery. As high-throughput techniques for molecular profiling continue to be developed and become more feasible, modeling these aspects can provide a deeper understanding of how perturbations spread in a heterogeneous biological interaction network and their functional consequences.

## Methods

### Reconstruction of three layers of biological molecular networks

#### Reconstruction of gene regulatory network

A subset of 695 human transcription factor motifs, corresponding to 695 unique transcription factors, was curated from the list provided by an online library of transcription factors and their binding motifs^68^. For each of these 695 motifs, the entire hg19 genome was scanned using a program that scans sequence databases to find occurrences of known motifs^69^, and significant hits with *p* < 1*e* − 3 were retained; 694 of the motifs had at least one significant hit in the genome for this scan. Once the genome-wide scan was completed, we took hg19 RefSeq annotated transcription start sites (TSS) and selected all associated Gene Symbols that mapped to a unique TSS. We then took the locations of the motif hits from the FIMO (Find Individual Motif Occurrences)^70^ scan described above and found the distance from the middle of the motif to the nearest TSS. Finally, we queried each of these files to find only motif-hits that occur in the promoter, which we defined as [−1000, +500] around the TSS. We used a *p* value cutoff of 1e−6 for the regulatory network layer of our multilayer network, which results in 18,566 nodes and 65,310 links.

In addition to the general gene regulatory network described above, we downloaded three additional tissue-specific gene regulatory networks from http://regulatorycircuits.org. In particular, we downloaded the set of cell-type and tissue-specific networks derived using data from the FANTOM5 project^18,47^. Of these, we selected networks in the smaller Network compendium dataset, which included three tissue-specific regulatory networks, forebrain (ID:03), lymphocytes (ID: 12), and lung (ID: 23). Based on the description in ref. ^18^, these networks are reconstructed by integrating CAGE-sequencing data with expression data. We then transformed these three tissue-specific networks by setting a link weight threshold of 0.05.

#### Construction of protein–protein interaction network

As our protein–protein interaction network layer, we used the comprehensive human interactome built by Cheng et al.^48^, which integrated multiple databases with experimental evidence. We mapped Entrez IDs to Gene Symbols using the HUGO Gene Nomenclature Committee (HGNC) website (https://www.genenames.org/). After removing self-loops, the resulting PPI network consisted of 15,930 proteins that were interconnected by 213,887 physical interactions. In this work, we focus on the largest (giant) connected component of the PPI network, which consists of 15,906 proteins connected by 213,874 links.

#### Construction of the metabolic network

For the metabolic network, we used the STITCH database^49^, which is an extensive association database that has both biochemical–biochemical (metabolite–metabolite) and biochemical-protein links. PubChem ids are used for metabolite identification, which maps well to metabolites in the Human Metabolome Database (HMDB)^50^, facilitating their identification. We limited our use of the dataset to interactions with experimental, similarity, and database evidence. The resulting metabolic association network, which we construct by combining the STITCH and HMDB databases, contains 1292 metabolites with HMDB ids and 16,032 interactions between them, and its largest (giant) connected component includes 1269 metabolites and 16,019 links. Compared to metabolic network layers built using Recon, the STITCH-based metabolic network contains a larger number of unique PubChem compounds as well as greater coverage of the PPI network via interlayer edges (see Table S2).

#### Compilation of metabolism-related gene lists

Single-nucleotide polymorphisms (SNPs) associated with lipid traits, blood pressure traits, and type 2 diabetes were compiled from previous large-scale GWAS published through 2016 from the literature, as well as the GWAS catalog. For lipid traits, SNPs from GWAS associated with low-density lipoprotein (LDL), very LDL, high-density lipoprotein, and total cholesterol levels were included. For blood pressure traits, SNPs associated with systolic pressure, diastolic pressure, mean arterial pressure, and pulse pressure values were selected. Type 2 diabetes loci included those associated with T2D, insulin secretion, and insulin resistance. Selection criteria included *p* values ≤ 1e−8 or Bonferroni corrected significance levels from European population-based studies. From these SNPs, gene lists were created based on either location (exon, intron, or promoter) or physical proximity to the nearest gene upstream or downstream of the SNP.

### Evaluation of the significance of the overlap between top influential genes and MC, MNC, and CNM gene sets

We estimated the significance of the overlap between the most influential genes and the disease-related gene sets (MC, MNC, and CNM) by evaluating the hypergeometric test *p* value of the overlap between each disease gene set and the top 500 influential genes. The results are shown in Fig. S9. Note that the hypergeometric test implicitly assumes as a null distribution a uniform sampling of the nodes in the network independent of their degree. To account for possible degree biases, we also evaluated a numerical *p* value by extracting three null distributions of 10,000 random gene sets with the same degree distribution as each disease gene set (MC, MNC, and CNM). We generated a random gene set as follows. We first chose a binning of the degree values of the network in order to avoid having singleton degree values. The binning is chosen such that each bin is populated by at least 30° values. Given a gene to randomize, we randomly extracted a new gene in the pool of genes whose degree falls in the same bin. We repeated this operation for each gene in the gene set to randomize and repeated the whole process 10,000 times. The significance of the overlap between the top influential genes and the disease gene set is estimated by comparing the observed overlap size with the null distribution of overlaps between the influential gene set and each random gene set. We repeated this operation for each disease gene set (MC, MNC, and CNM) and each gene regulatory network variant (general and three tissue-specific networks). In Fig. S10, we show the empirical *p* values computed in each case. Note that for visualization purposes we assigned an arbitrary value of 1e−6 to the *p* values that were below the resolution allowed by the considered number of samples. We observe in both cases that influential genes are enriched in complex-disease-related genes, while this enrichment is not significant or marginally significant in the case of Mendelian disease genes. We hypothesize that this trend stems from the fact that complex diseases are the result of the interaction between a large number of units, and, therefore, are more likely to be associated with genes that are well connected in the network.

### A theoretical framework for analyzing the robustness of multilayer molecular networks

We developed a general theoretical framework for modeling cascading failures between the gene regulatory and PPI networks, and computing the final number of functional nodes in three molecular layers after randomly removing a 1 − *p* fraction of genes from the gene regulatory network. We find that the gene regulatory network is vulnerable to perturbations, while the robustness of the metabolic network is highly dependent upon support from the PPI network.

#### Percolation analysis in single-gene regulatory networks

We denote the joint degree distribution of the top-layer gene regulatory network as *P*_Gene_(*k*_in_, *k*_out_). We randomly choose a fraction 1 − *p* of nodes as perturbed genes. The probability density of genes with in-degree *k*_in_ and out-degree *k*_out_ not being perturbed or TGs of one perturbed gene is ${P}_{{\rm{Gene}}}({k}_{{\rm{in}}},{k}_{{\rm{out}}}){p}^{{k}_{{\rm{in}}}+1}$. Thus, after removing the perturbed genes and their targets, the fraction of the remaining nodes is

1 $${r}_{{\rm{S}}}=\mathop{\sum }\limits_{{k}_{{\rm{out}} = 0}}^{\infty }\mathop{\sum }\limits_{{k}_{{\rm{in}}}=0}^{\infty }{P}_{{\rm{Gene}}}({k}_{{\rm{in}}},{k}_{{\rm{out}}}){p}^{{k}_{{\rm{in}}}+1}.$$

Assuming that there are no correlations between the in-degrees and out-degrees, the degree distribution of the remaining network can be written as

2 $${P}^{({r}_{{\rm{S}}})}({k}_{{\rm{in}}},{k}_{{\rm{out}}})=\mathop{\sum }\limits_{i\ge {k}_{{\rm{in}}}}^{\infty }\mathop{\sum }\limits_{j\ge {k}_{{\rm{out}}}}^{\infty }{P}_{\rm{Gene}}(i,j)\left(\begin{array}{l}i\\ {k}_{\rm{in}}\end{array}\right)\left(\begin{array}{l}j\\ {k}_{\rm{out}}\end{array}\right){r}_{{\rm{S}}}^{{k}_{{\rm{in}}}}{(1-{r}_{{\rm{S}}})}^{i-{k}_{{\rm{in}}}}{r}_{{\rm{S}}}^{{k}_{{\rm{out}}}}{(1-{r}_{{\rm{S}}})}^{j-{k}_{{\rm{out}}}},$$

where $$\left(\begin{array}{l}i\\ k\end{array}\right)=\frac{i!}{k!(i-k)!}$$ is a combination. In the remaining network, the isolates fail and the functional network size is

3 $${f}_{{\rm{S}}}^{{\rm{G}}}={r}_{{\rm{S}}}\left[1-\mathop{\sum }\limits_{i\ge 0}^{\infty }\mathop{\sum }\limits_{j\ge 0}^{\infty }{P}_{\rm{Gene}}(i,j){(1-{r}_{{\rm{S}}})}^{i}{(1-{r}_{{\rm{S}}})}^{j}\right],$$

which can be simplified to

4 $${f}_{{\rm{S}}}^{{\rm{G}}}={r}_{{\rm{S}}}[1-{P}^{({r}_{{\rm{S}}})}({k}_{{\rm{in}}}=0,{k}_{{\rm{out}}}=0)],$$

We apply this theoretical tool to gene regulatory networks, including a generic network and three tissue-specific networks. As shown in Fig. S2, the theoretical predictions (solid lines) agree well with the simulations (symbols).

#### Percolation analysis in coupled gene regulatory, PPI, and metabolic networks

Owing to the incompleteness of the data, some proteins do not have corresponding genes in the regulatory networks, and some genes do not have corresponding proteins in the PPI network. Thus, the gene regulatory and the PPI networks are partially interdependent, and their interdependency relations are not random. We propose a method to find the equivalent coupling strengths between the gene regulatory and PPI networks, denoted by *q*_G_ and *q*_P_, so that the non-randomly interdependent relations could be approximated by random interdependency (see Supplementary Note 2).

We present the solution of the final functional network sizes step-by-step according to the cascading process between the gene regulatory and the PPI networks. In order to unify the quantities at each stage *t* of the cascading process, we define ${\psi }_{t}^{\prime}$ as the remaining network size after initial perturbation or upon receiving the failure from the PPI network, and *ψ*_*t*_ as the functional network size in the gene regulatory network. At the initial stage *t* = 1, the remaining network size after perturbation is ${\psi }_{1}^{\prime}=p$, and the functional network is ${\psi }_{1}={\psi }_{1}^{\prime}{h}_{{\rm{Gene}}}({\psi }_{1}^{\prime})$, where ${h}_{{\rm{Gene}}}(p)={r}_{{\rm{S}}}/p[1-{P}^{({r}_{{\rm{S}}})}({k}_{{\rm{in}}}=0,{k}_{{\rm{out}}}=0)]$. Since a fraction of *q*_P_ nodes in the PPI network depends on nodes from the gene regulatory network, the number of nodes in the PPI network becoming dysfunctional is $(1-{\psi }_{1}){q}_{{\rm{P}}}={q}_{{\rm{P}}}(1-{\psi }_{1}^{\prime}{h}_{{\rm{Gene}}}({\psi }_{1}^{\prime}))$. Accordingly, the remaining network size in the PPI network is ${\phi }_{1}^{\prime}=1-{q}_{{\rm{P}}}(1-{\psi }_{1}^{\prime}{h}_{{\rm{Gene}}}({\psi }_{1}^{\prime}))$. In the PPI network, the generating functions for the degree distribution and branching process are, respectively, ${G}_{{\rm{PPI}}}(x)=\mathop{\sum }\nolimits_{k = 0}^{\infty }{P}_{{\rm{PPI}}}(k){x}^{k}$ and ${H}_{{\rm{PPI}}}(x)=G^{\prime} (x)/G^{\prime} (1)$. The fraction of nodes belonging to the largest connected component in the PPI is ${\phi }_{1}={\phi }_{1}^{\prime}{h}_{{\rm{PPI}}}({\phi }_{1}^{\prime})$, where *h*_PPI_(*p*) = 1 − *G*_PPI_(*px*_*c*_ + 1 − *p*) with *x*_*c*_ = *H*_PPI_(*px*_*c*_ + 1 − *p*). Following this approach, we can construct the sequence for the remaining network sizes ${\psi }_{t}^{\prime}$ and ${\phi }_{t}^{\prime}$, and the functional network sizes *ψ*_*t*_ and *ϕ*_*t*_. The general form is given by

5 $$\psi ^{\prime}_{1} = \, p,\quad {\psi }_{1}=\psi ^{\prime}_{1} {h}_{{\rm{Gene}}}({\psi }_{1}^{\prime})\\ \phi ^{\prime}_{1} = \, 1-{q}_{{\rm{P}}}(1-{h}_{{\rm{Gene}}}({\psi }_{1}^{\prime})p),\quad {\phi }_{1}={\phi }_{1}^{\prime}{h}_{{\rm{PPI}}}({\phi }_{1}^{\prime})\\ {\psi }_{2}^{\prime}= \, p(1-{q}_{{\rm{G}}}(1-{h}_{{\rm{PPI}}}({\phi }_{1}^{\prime}))),\quad {\psi }_{2}={\psi }_{2}^{\prime}{h}_{{\rm{Gene}}}({\psi }_{2}^{\prime})...,\\ {\psi }_{n}^{\prime}= \, p(1-{q}_{{\rm{G}}}(1-{h}_{{\rm{PPI}}}({\phi }_{n-1}^{\prime}))),\quad {\psi }_{n}={\psi }_{n}^{\prime}{h}_{{\rm{Gene}}}({\psi }_{n}^{\prime})\\ {\phi }_{n}^{\prime}= \, 1-{q}_{{\rm{P}}}(1-{h}_{{\rm{Gene}}}({\psi }_{n}^{\prime})p),\quad {\phi }_{n}={\phi }_{n}^{\prime}{h}_{{\rm{PPI}}}({\phi }_{n}^{\prime}).$$

At the end of the cascading process, no further failures occur. The remaining fractions of nodes in the gene regulatory network and the PPI reach stable values, ${\psi }_{m}^{\prime}={\psi }_{m+1}^{\prime}$ and ${\phi }_{m}^{\prime}={\phi }_{m+1}^{\prime}$, respectively. Thus, the fractions of the final functional nodes in the regulatory and PPI networks are, respectively, ${f}_{{\rm{S}}}^{{\rm{G}}}={\psi }_{m}$ and ${f}_{{\rm{S}}}^{{\rm{P}}}={\phi }_{m}$.

The PPI and the metabolic networks are connected by multiple unidirectional support-dependence relations. In the metabolic network, *q*_Meta_ is the fraction of metabolites having multiple connections with the PPI layer. The generating functions of the degree distribution of the metabolic network and its branching process are ${G}_{{\rm{Meta}}}(x)=\mathop{\sum }\nolimits_{k = 0}^{\infty }{P}_{{\rm{Meta}}}(k){x}^{k}$ and ${H}_{{\rm{Meta}}}(x)={G}_{{\rm{Meta}}}^{\prime}(x)/{G}_{{\rm{Meta}}}^{\prime}(1)$, respectively. Each metabolite has *k*_*s*_ supporting links from the PPI network, and we define the support degree distribution as *P*_D_(*k*_*s*_) whose generating function is ${G}_{{\rm{D}}}(x)=\mathop{\sum }\nolimits_{{k}_{s} = 0}^{\infty }{P}_{{\rm{D}}}({k}_{s}){x}^{{k}_{s}}$, and whose branching process is ${H}_{{\rm{D}}}(x)={G}_{{\rm{D}}}^{\prime}(x)/{G}_{{\rm{D}}}^{\prime}(1)$.

Since failure in the metabolic network cannot affect the gene regulatory and PPI networks, their percolation behaviors are equivalent to that in the coupled gene regulatory and PPI networks, whose final number of functional nodes are *ψ*_*m*_ and *ϕ*_*m*_, respectively. In the metabolic network, a metabolite node fails if more than *f*_P2M_ fraction of its supporting proteins fails. The probability that more than *f*_P2M_ fraction of the supports to a metabolite fails is $$\omega ={q}_{{\rm{Meta}}}\mathop{\sum }\nolimits_{{k}_{s} = 0}^{\infty }{P}_{{\rm{D}}}({k}_{s})\mathop{\sum }\nolimits_{l = \lceil {f}_{{\rm{P}}2{\rm{M}}}{k}_{s}\rceil }^{{k}_{s}}\left(\begin{array}{l}{k}_{s}\\ l\end{array}\right){(1-{\phi }_{m})}^{l}{\phi }_{m}^{{k}_{s}-l}$$. Thus, the fraction of the remaining nodes is *r*_Meta_ = 1 − *ω*, and the final number of a functional node of the metabolic network is

6 $${f}_{{\rm{S}}}^{{\rm{M}}}={r}_{{\rm{Meta}}}(1-{G}_{{\rm{Meta}}}({r}_{{\rm{Meta}}}{x}_{c}+1-{r}_{{\rm{Meta}}})),\quad {x}_{c}={H}_{{\rm{Meta}}}({r}_{{\rm{Meta}}}{x}_{c}+1-{r}_{{\rm{Meta}}}).$$

### Reporting summary

Further information on research design is available in the Nature Research Reporting Summary linked to this article.

## Supplementary information

Supplementary Information

Reporting Summary

## Data Availability

The data used in this work are collected from open-access databases. The transcription factors and their DNA binding motifs used in the construction of the human regulatory network were downloaded from the CIS-BP database (http://cisbp.ccbr.utoronto.ca/). The three tissue-specific gene regulatory networks were downloaded from the FANTOM5 database^47^, respectively forebrain (ID: 03), lymphocytes (ID: 12), and lung (ID: 23). The PPI network data were obtained from Supplementary Data 1 provided by Cheng et al.^48^. The human chemical–chemical (chemical_chemical.links.detailed.v4.0.tsv) and protein–biochemical (9606.protein_chemical.links.detailed.v4.0.tsv) links were downloaded from the STITCH database (version 4.0) (http://stitch.embl.de/). The human metabolites data were downloaded from the Human Metabolome Database (HMDB) (version 3.5, retrieved November 2015). The essential and essential genes were respectively collected from the Database of Essential Genes (DEG)^56^ and the Cancer Gene Census (CGC)^57^. The metabolic disease-associated genes were integrated from large-scale genome-wide association studies (GWAS) published in the literature and data from the NHGRI-EBI GWAS Catalog (https://www.ebi.ac.uk/gwas/)^65^. The authors declare that the data supporting the findings of this study are available from the corresponding author upon request.

## Code availability

The main results are generated by using Matlab-R2017a, R-3.6.1, and python 2.7. The code generating the main results in this work is available at https://github.com/XuemingLiu/Robust-Multilyer-BioNetworks.
