# Supplementary material for: Robustness and lethality in multilayer biological molecular networks
Source: Nat Commun. 2020 Nov 27;11:6043. doi: 10.1038/s41467-020-19841-3 (PMC7699651; doi:10.1038/s41467-020-19841-3)
Supplement: Supplementary file 1 — Supplementary Information [file 41467_2020_19841_MOESM1_ESM.pdf]

Supplementary Information: Robustness and lethality in multilayer biological molecular networks

Xueming Liu[1,5*], Enrico Maiorino[2,5], Arda Halu[2,5], Kimberly Glass[2], Rashmi B. Prasad[3], Joseph Loscalzo[2], Jianxi Gao[4*], Amitabh Sharma[2*]

[1] *Key Laboratory of Imaging Processing and Intelligent Control, School of Artificial Intelligence and Automation, Huazhong University of Science and Technology, Wuhan 430074, China*

[2] *Channing Division of Network Medicine, Department of Medicine, Brigham and Women's Hospital, Harvard Medical School, Boston, MA 02115, USA*

[3] *Genomics Diabetes and Endocrinology, Lund University Diabetes Centre, CRC, Malmö 21429*

[4] *Department of Computer Science, Rensselaer Polytechnic Institute, Troy, New York, 12180, USA*

[5] *These authors contributed equally: Xueming Liu, Enrico Maiorino, Arda Halu.*

∗ *email: xm_liu@hust.edu.cn; gaoj8@rpi.edu; reash@channing.harvard.edu*

(Dated: October 12, 2020)

Supplementary Note 1. Dynamic process in multilayer biological molecular networks after perturbations of genes

After initial perturbations of the genes in the gene regulatory network, the perturbed genes and their target genes become dysfunctional, and then the resulting isolates (i.e. nodes without any edges) become dysfunctional. The corresponding proteins of the dysfunctional genes become dysfunctional, fragmenting the PPI and causing the proteins that are not in the largest connected component to become dysfunctional. The failure of newly dysfunctional proteins will be further reflected to the gene regulatory network as an additional perturbation. This process will iterate, until until no more genes or proteins are removed from the system. Ultimately, the failure spreads to metabolic layer. Metabolites fail if more than f_{P2M} fraction of supporting proteins fail, fragmenting the metabolic network; the metabolites that do not belong to the largest connected component also become dysfunctional. Figure S1 shows the flow of the dynamic iterative process.

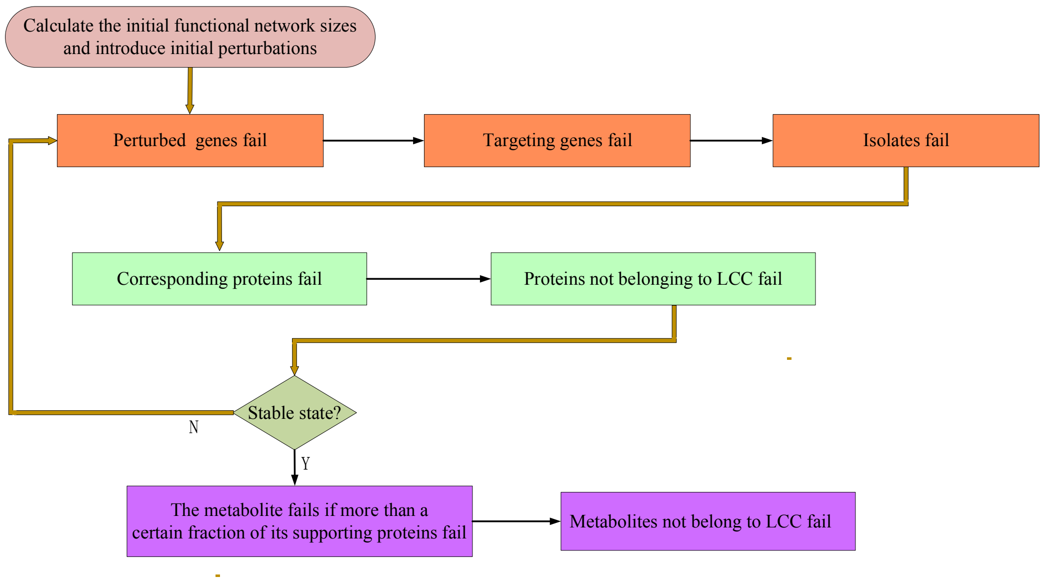

FIG. S1. Dynamic process of spreading failure

The process of cascading failure proposed in our work shares several similarities with a perturbation process proposed recently by Klosik et al. [1]. In their work, however, the authors distinguish the links in two classes by assigning them a logical function, the "AND" or "OR" operators. In this setting, the failure of a node is caused by at least one neighbor failing in the case of AND links, and by all the neighbors failing in the case of OR

links. Applying this description, our process corresponds to treating all the links in the gene regulatory network as "AND" operators and all the protein-metabolite interlayer links as thresholded "OR" operators.

Supplementary Note 2. Calculating the equivalent coupling strengths between gene regulatory and PPI networks

Genes in the gene regulatory network and proteins in the PPI network form one-to-one interdependent pairs. Owing to the incompleteness of the data, the gene regulatory and PPI networks are only partially interdependent of each other. We use N_{G} and N_{P} to denote the numbers of nodes in the gene regulatory and PPI networks, respectively, and N_{I} denotes the number of gene-protein pairs. If the interdependencies between the gene regulatory and PPI networks are random in topology without any correlations in the degrees of the connected gene-protein pairs, then the coupling strengths of the regulatory and PPI networks are $q_{\mathrm{G}}^{\mathrm{Rand}} = N_{\mathrm{I}}/N_{\mathrm{G}}$ and $q_{\mathrm{P}}^{\mathrm{Rand}} = N_{\mathrm{I}}/N_{\mathrm{P}}$. In the real network cases, the connections between genes and proteins are not random, leading to a situation in which the coupling strengths are difficult to quantify. To solve this problem, we propose a method for finding equivalent coupling strengths so that the couplings between the regulatory and PPI networks can be treated as random in theoretical computations.

In the gene regulatory network, the fraction of genes that do not have corresponding proteins is $p_{\mathrm{GI}} = (N_{\mathrm{G}} - N_{\mathrm{I}})/N_{\mathrm{G}}$, and these nodes form a sub-network with no connections to the PPI network. We refer to this sub-network as an independent regulatory sub-network. Treating the independent regulatory sub-network as an isolated network and given its degree distribution, $P_{\mathrm{GI}}(k_{\mathrm{in}}, k_{\mathrm{out}})$, we can estimate the final functional node size $f_{\mathrm{S}}^{\mathrm{GI}}$ in the independent regulatory sub-network by substituting $P_{\mathrm{GI}}(k_{\mathrm{in}}, k_{\mathrm{out}})$ with $P_{\mathrm{Gene}}(k_{\mathrm{in}}, k_{\mathrm{out}})$ in Eqs. (1) and (3) (see main text). As a result, the final functional node size is $p_{\mathrm{GI}} * f_{\mathrm{S}}^{\mathrm{GI}}$ if only the nodes in the independent regulatory sub-network remain, which is equal to the case of intentionally perturbing all of the genes that are connected to the PPI network. If we convert such a perturbation strategy into random perturbations, how many nodes are required to be perturbed in the isolated gene regulatory network so that the final functional node size is $p_{\mathrm{GI}} * f_{\mathrm{S}}^{\mathrm{GI}}$? Figure S2 shows mappings from random perturbations on $1 - p$ fraction of genes to the functional node sizes. With such mapping, we could find an equivalent random

perturbation fraction $1 - p_{eG}$, having the final functional node size $p_{GI} * f_S^{GI}$. Thus, the equivalent coupling strength from the gene regulatory network to the PPI network could be approximated by $q_G = 1 - p_{eG}$.

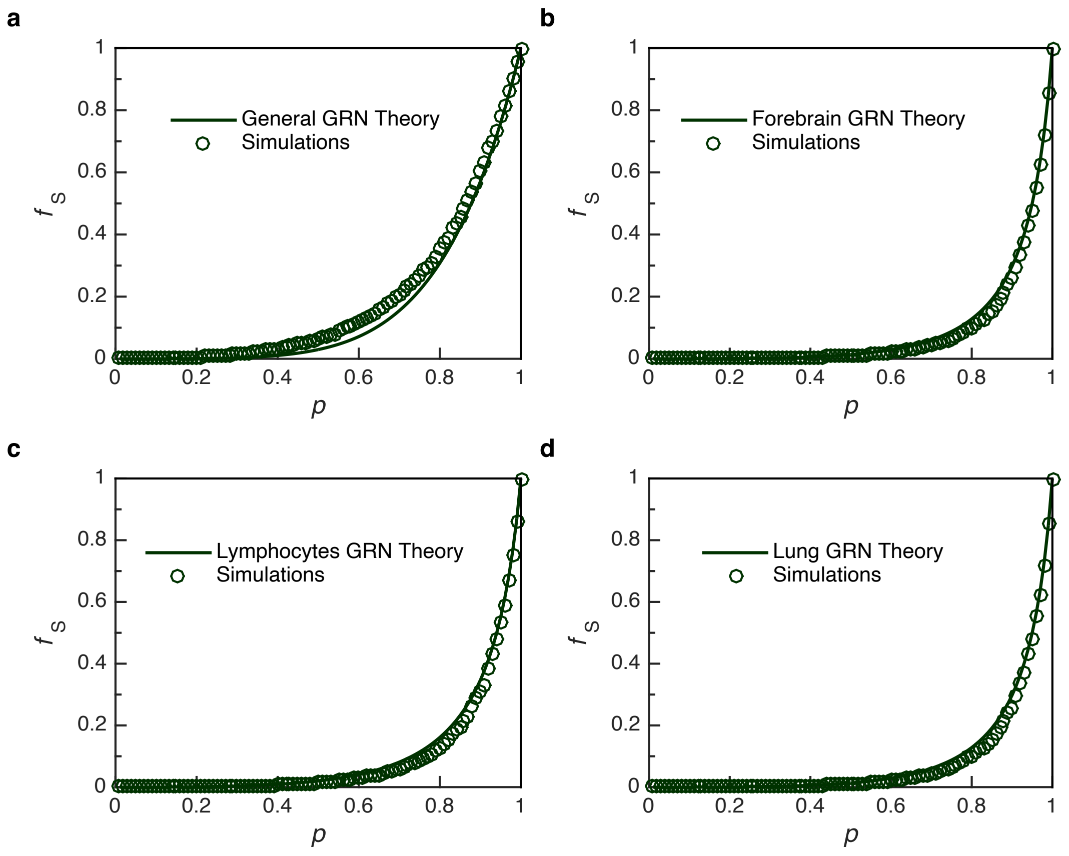

FIG. S2. The size of the final functional genes after randomly perturbing $1 - p$ fraction of genes in the isolated general gene regulatory network (**a**), and in the isolated tissue-specific networks of forebrain (**b**), lymphocytes (**c**), and lung (**d**). The theoretical predictions (solid lines) agree well with the simulations (symbols, averaged over 30 realizations), confirming our theoretical analysis.

Similarly, treating the independent PPI sub-network as an isolated network and given its degree distribution, $P_{PI}(k)$, the giant connected component size is $W_{PI} = 1 - G_{PPI}(x_c) = 1 - \sum_{k=0}^{\infty} P_{PI}(k) x_c^k$ where $x_c = H_{PPI}(x_c) = G'_{PPI}(x_c)/G'_{PPI}(1)$. Based on mapping of the fraction p of remaining nodes to the size of the giant connected component in the isolated PPI, as shown in Figure S3, we can find a sub-network formed by a fraction p_{eP} of randomly chosen nodes with its giant connected component being $W_{PI} * (N_P - N_I)/N_P$. The equivalent coupling strength from the PPI to the gene regulatory network is $q_P = 1 - p_{eP}$.

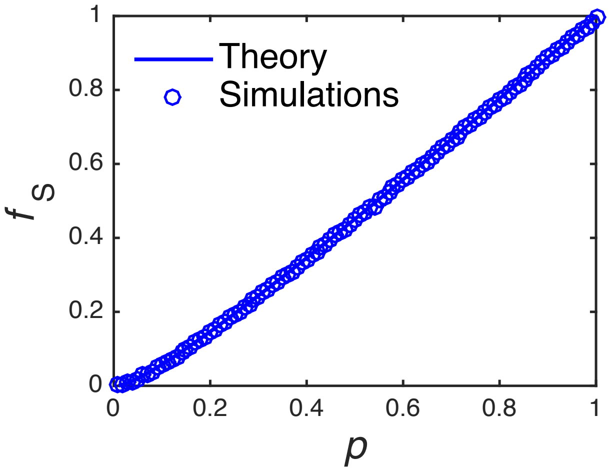

FIG. S3. The size of the giant connected component of the sub-network formed by the randomly chosen p fraction of proteins. The solid line shows the theoretical predictions, which agree well with the simulation results (symbols, averaged over 30 realizations).

TABLE S1. **The statistical correlations between the thoery and simulation results**

	General GRN	Forebrain GRN	Lymphocytes GRN	Lung GRN
Gene regulatory layer	0.9948	0.9893	0.9908	0.9910
PPI layer	0.9938	0.9960	0.9966	0.9968
Metabolic layer	0.9813	0.9879	0.9915	0.9913

Supplementary Note 3. Applying the theoretical framework to multilayer erdős-rényi networks

We apply the theoretical framework to multilayer ER networks which models the failure mechanisms in the multilayer biological molecular networks, in order to show the generality of our framework. In the multilayer ER networks, a directed ER network is partially interdependent with an undirected ER network by one-to-one correspondence, and such an undirected ER network give multiple supports to another undirected ER network. Figure S4 shows the solution of the final functional node sizes of three coupled ER networks after randomly removing $1 - p$ fraction of nodes in the directed ER network. The solid lines are the theoretical predictions (solid lines), which agree well with the simulation results (symbols).

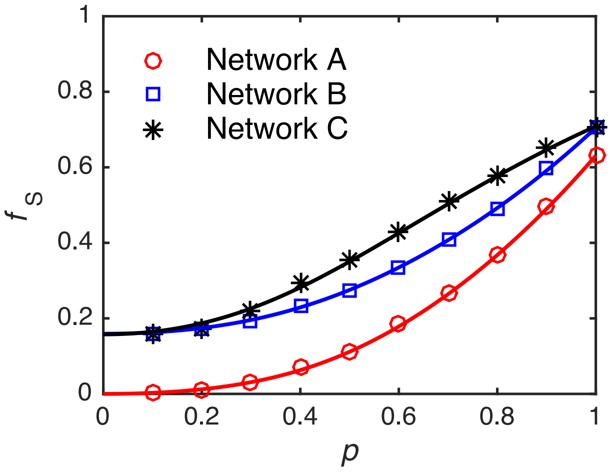

FIG. S4. The final functional node sizes in the coupled ER networks, after randomly removing $1-p$ fraction of nodes from the directed ER network (Network A with $\langle k \rangle = 1$). Network A is interdependent with Network B ($\langle k \rangle = 10$) having a coupling strength of 0.8. A fraction of 0.8 nodes in Network C ($\langle k \rangle = 3$) has multiple supports from Network B, with the average degree of the multiple supports of 2. The solid lines represent the theoretical predictions, and the symbols represent the simulation results, which are averaged over 30 realizations. The Pearson correlation coefficient between the theory and simulation results for Network A, B and C are respectively 1, 0.9999 and 0.9997, indicating a great agreement between them.

Supplementary Note 4. Essential and cancer genes enrichment and validation

To measure the enrichment of a set of genes in the ranking obtained from the influence scores, we identify the considered genes as the true positives of a classification problem. The influence score of a gene is thus analogous to the confidence of our model in assigning that gene to the positive class. Under this perspective, the enrichment of the influential genes can be evaluated with standard statistical tools. In this work, we considered the precision-recall curve as a meaningful performance metric in virtue of its robustness to class imbalance. The performance associated with each precision-recall curve is evaluated through the average precision score (APS), a score defined as

$$\text{APS} = \sum_n (R_n - R_{n-1}) P_n$$

where P_n and R_n are the precision and recall at the n-th threshold. A large value of APS indicates that the considered genes are more likely to have high influence scores. This

evaluation was performed on essential and cancer genes.

To uncover the mechanism that the essential and cancer genes are enriched in the genes with high influence scores, we calculate the number of genes becoming dysfunctional in the second round of the cascading process. The removal of each gene could cause its corresponding protein and the proteins that apart from the largest connected component losing its function, which is called the first round in the cascading failure process. If failure in the PPI network goes back to the gene regulatory network, more genes stops functioning, which could cause more proteins becoming dysfunctional. This is called the second round in the cascading failure process. We find that the removal of essential and cancer could cause more genes and proteins becomes dysfunctional in the second round, than the removal of non-essential and non-cancer genes, as shown in the Fig. 2c and 2d in the main text.

There are different definitions of essential genes. At the individual level, a gene can be defined as essential if its loss of function will lead to individual death or loss of fitness. At the population level, a gene is essential if the biological system shows intolerance to loss-of-function variants [2]. Next, we will show that genes important to a system's robustness are enriched in genes with high scores of essentiality valued by different metrics. We choose three additional sets of essential genes measured by: (1) the probability of haploinsufficiency (Phi), (2) the probability of loss-of-function intolerance (pLI), and (3) the essential genes found by Dickinson et al. [3]. As Figure S7 shows, the result holds for the essential genes valued by these three different metrics. Since that the metrics for essentiality are highly correlated with one another [3], we can infer that this results also holds for other metrics not tested here.

Supplementary Note 5. Comparison with genome-scale metabolic models

For a quantitative comparison of the STITCH-based and Recon-based metabolic networks, we built metabolite networks from Recon 1, Recon 2 and Recon 3D using the Python implementations of the COBRA Toolbox [4, 5]. We mapped the metabolite BiGG IDs to PubChem CIDs in two steps: First by matching the BiGG IDs with their corresponding InChI keys and then using the PubChem Identifier Exchange Service to match the InChI keys with PubChem compound CIDs. The number of corresponding metabolites and CIDs in each model is summarized in Table S2. Since metabolites in Recon models include multi-

TABLE S2. **Summary of the quantitative comparison between the STITCH- and Recon-based metabolite networks.**

	#Genes	#Metabolites	#Metabolites (Unique CIDs)	#Common metabolites with STITCH	#Overlapping edges with STITCH (two-sided Fisher's exact p-value and odds-ratio)
STITCH	12,039	1,292	1,292		
Recon 1	1,905	2,766	686	108	142 (p=1.97e-59, OR=9.94)
Recon 2	2,140	5,063	825	96	90 (p=2.27e-44, OR=11.05)
Recon 3D	2,248	5,835	836	126	275 (p=2.68e-146, OR=15.34)

ple compartments that map to the same chemical compound, the overall metabolite coverage of the STITCH-based metabolite network was higher than that of Recon in terms of the unique compounds to which the metabolites mapped. To assess fairly the agreement of the two networks, we focused on the edge overlap between the networks formed by the set of metabolites common to STITCH and Recon networks. In all Recon models tested, there was a statistically significant enrichment of overlapping edges (see Table S2). Notably, the number of overlapping edges was markedly increased in the latest version, Recon 3D, suggesting that STITCH networks successfully capture the improvements in the subsequent versions of Recon models and hinting at the gradual convergence of these datasets.

To compare further our approach with alternative methods that identify nodes with impact on robustness of biological systems, we used an established *in silico* single gene deletion approach based on flux balance analysis (FBA) [6–8] in which the effect of the single gene knockout on the objective value (typically the biomass, also used here as the sole objective value) is simulated. In this framework, one can keep track of the impact of the single gene knockout by optimizing the objective function post-knockout and comparing it with the original objective value. A gene is considered essential if restricting the flux of all reactions that depend on it to zero causes the objective (i.e., the growth rate) also to be either zero or below a given viability threshold. This method also applies in the same way to the deletion of reactions from the metabolic network, resulting in the identification of essential reactions. Using Python implementations of the COBRA Toolbox [4, 5], we performed this analysis for the Recon3D human metabolic model. We found that in Recon3D, only one gene (BiGG ID: 54675_AT1) is truly essential, resulting in a complete depletion of metabolic fluxes with its removal (Figure S21). In addition, we kept a record of all other genes whose deletion results in some decrease in flux in the biomass equation. We found 39 additional such genes, but since the impact of their deletion on the biomass was much smaller in comparison

(< 20% of unperturbed value), they would not conventionally be deemed essential (Figure S21). The rest of the $> 2,000$ genes in Recon3D had no impact on the biomass.

To ensure that this small number of essential genes by the FBA criteria is in agreement with other human metabolic models, we ran the same analysis for all of the 107 metabolic models in the BiGG database covering over 20 organisms. The Recon models (Recon1 and Recon3D), along with the two other human metabolic models (iAB_RBC_283 and iAT_PLT_636) in BiGG, consistently showed the most robust response to these single gene knockouts with a very small proportion of genes and reactions deemed essential by the above criteria. This finding is in contrast to many other organisms having as high as 4% and 2% of metabolic reactions and enzyme-encoding genes, respectively, flagged as essential (Figure S22).

Algorithm 1 Assortative randomizations on the gene regulatory network.

Input: $G(N, E)$ (the gene regulatory network)
Output: G' (randomized network).

$G' \leftarrow G$
Rank the nodes according to their in-degrees/out-degrees in a descending order to get node sequences I/O.
for $i \leftarrow 1$ to N **do**
  Select all the E_{I_i} links pointing to node I_i and all the E_{O_i} links pointing to node O_i
  for $j \leftarrow 1$ to E_{I_i} **do**
    if no multiple links or self loop will appear **then**
      rewire the link to point to node O_i
    end
  end
  for $k \leftarrow 1$ to E_{O_i} **do**
    if no multiple links or self loop will appear **then**
      rewire the link to point to node I_i
    end
  end
end

FIG. S5. Pseudo-code of generating randomization models of gene regulatory networks.

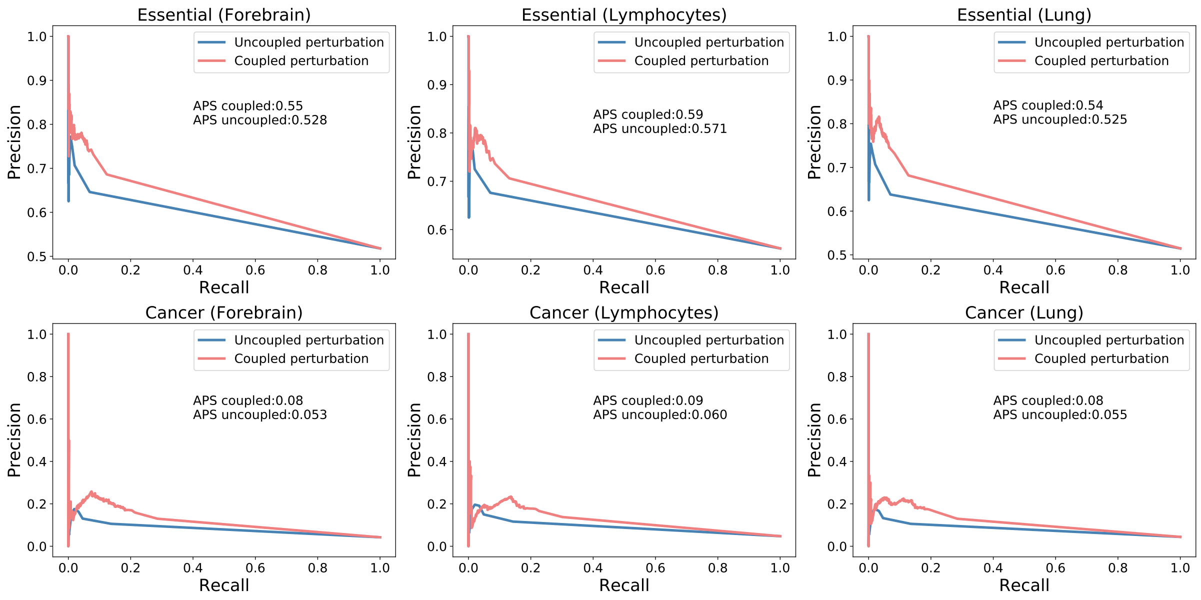

FIG. S6. Precision-recall curves based on the prioritization of essential genes (top row) and cancer genes (bottom row) in both the uncoupled (blue) and coupled (red) case for three tissue specific networks: forebrain (left column), lymphocytes (middle column), and lung (right column).

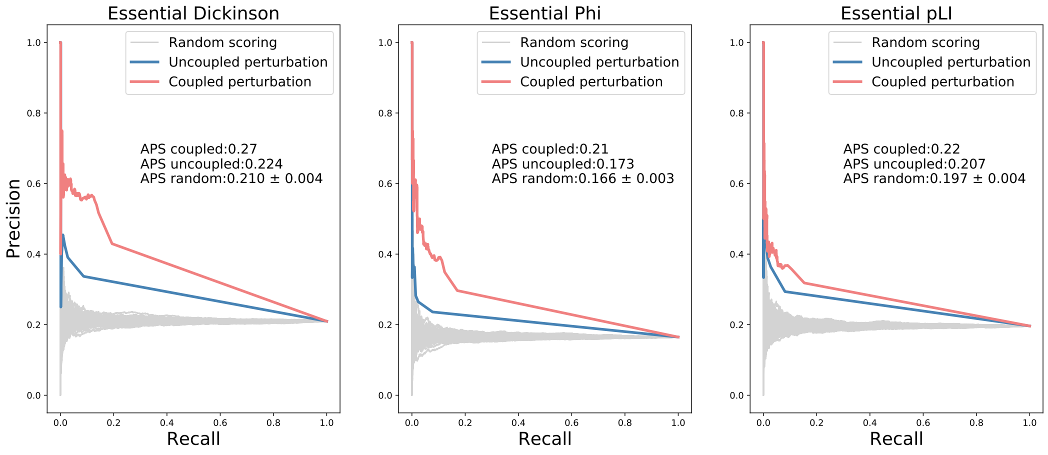

FIG. S7. Precision-Recall (PR) curves of the coupled (red) and uncoupled (blue) influence scores in the prioritization of essential genes valued by three other different metrics. The gray PR curves represent 100 random node rankings. On the right of each plot are listed the Average Precision Scores (APS) of the three ranking strategies evaluated from the corresponding PR curves. The APS scores in the coupled case are larger than the uncoupled and random (gray) cases.

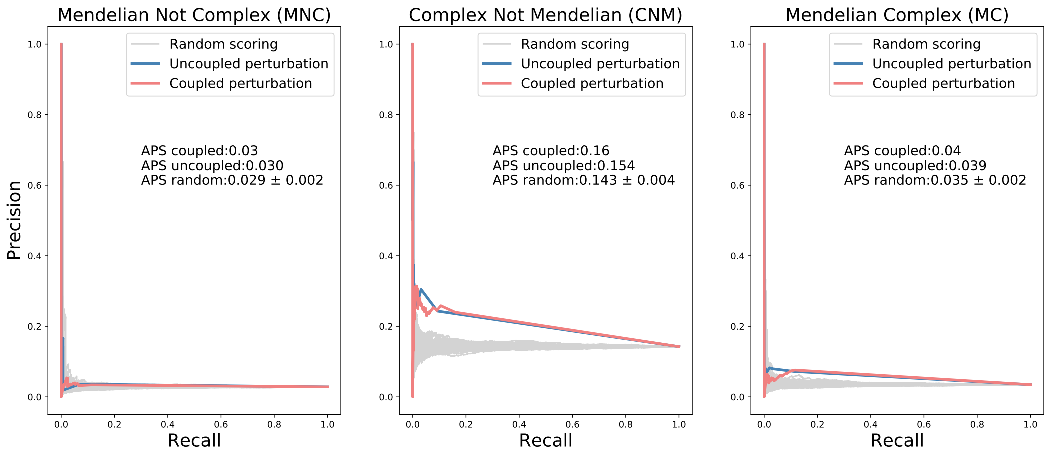

FIG. S8. Precision-Recall (PR) curves of the coupled (red) and uncoupled (blue) influence scores in the prioritization of MNC, CNM, and MC disease genes. The gray PR curves represent 100 random node rankings. On the right of each plot are listed the Average Precision Scores (APS) of the three ranking strategies evaluated from the corresponding PR curves.

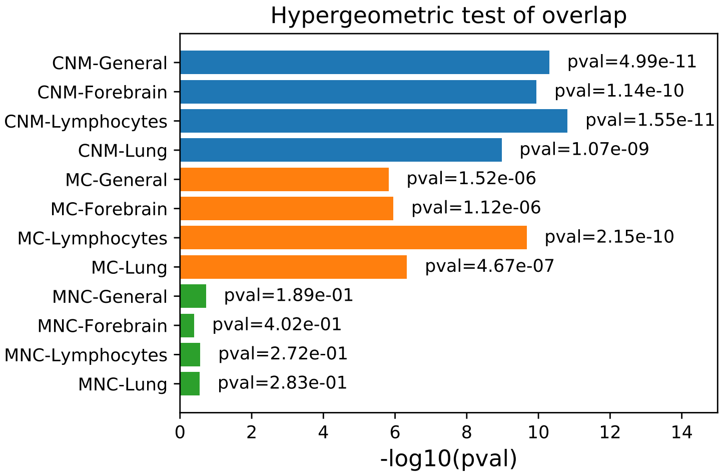

FIG. S9. Enrichment of Complex Not Mendelian (CNM), Mendelian Complex (MC) and Mendelian Not Complex (MNC) genes in top 500 influential genes. Bar length represents the -log(p-value) of the hypergeometric test of overlap between each gene set and the top 500 influential genes.

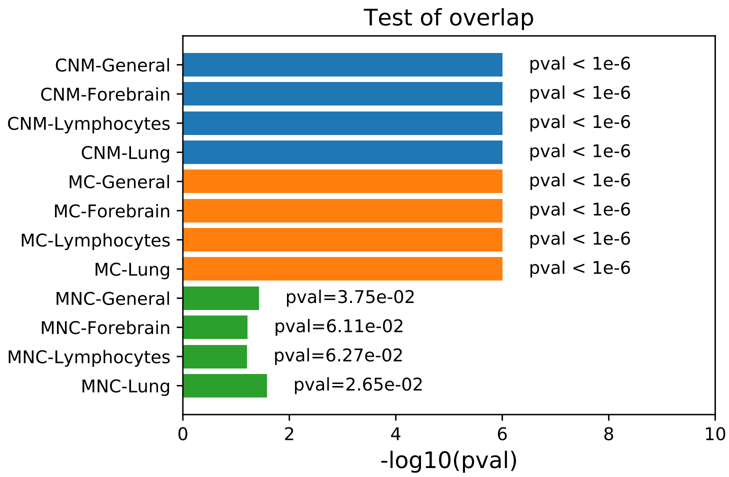

FIG. S10. Enrichment of Complex Not Mendelian (CNM), Mendelian Complex (MC) and Mendelian Not Complex (MNC) genes in top 500 influential genes. Bar length represents the -log(p-value) of the empirical p-value obtained by comparing the observed overlap to the null distribution of overlaps between each gene set and 10000 random gene sets. A conventional lower bound of 1e-6 has been chosen to represent p-values lower than the minimum numerical resolution.

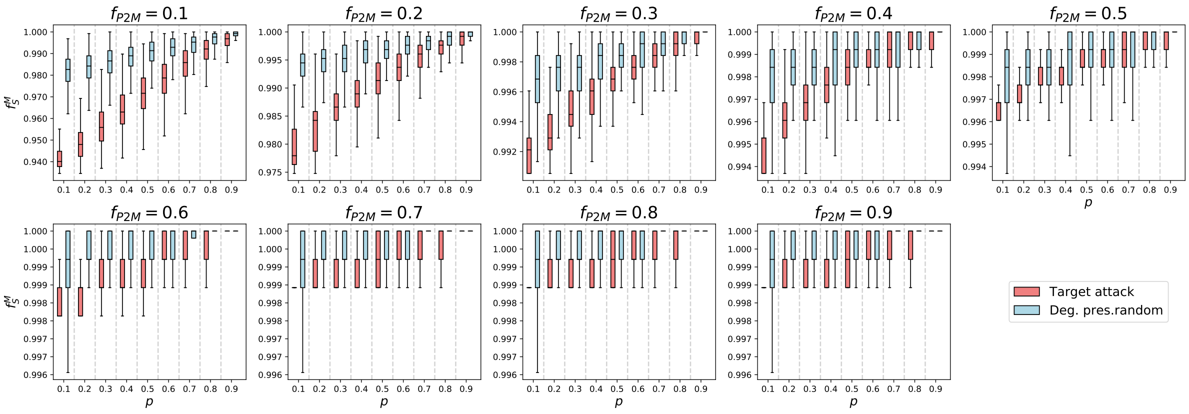

FIG. S11. Targeting dyslipidemia-related genes (red boxes) causes more damage to the metabolic network than degree-preserving random attacks (blue boxes). Fraction of functional nodes after perturbations for several values of remaining fraction p of metabolic disease genes as the threshold proportion f_{P2M} varies from 0.1 to 1. Lower values indicate a higher degree of damage to the network. The results are calculated based on 1000 random realizations. For each distribution, boxes indicate the quartiles, whiskers extend to an additional 1.5 * IQR interval, and the medians are represented by a black line.

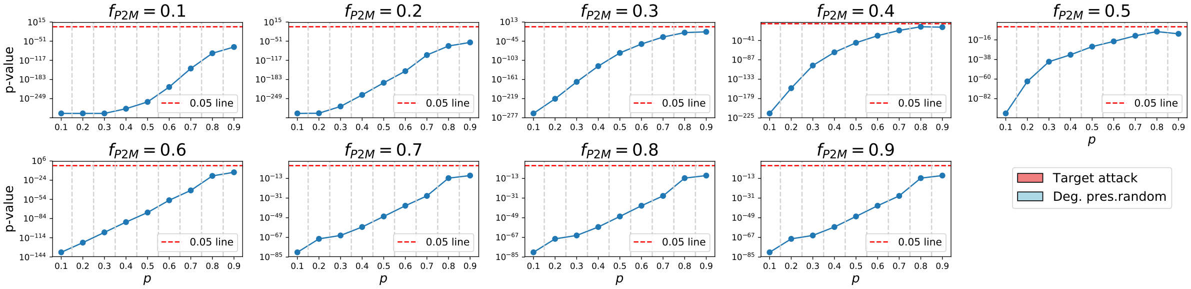

FIG. S12. Statistical analysis of the difference between the targeted attacks on dyslipidemia-related genes and degree-preserving random attacks. The curves represent the -log(p-value) scores of the one-sided Mann-Whitney test when comparing the two distributions of final functional network size for every value of p and f_{P2M}.

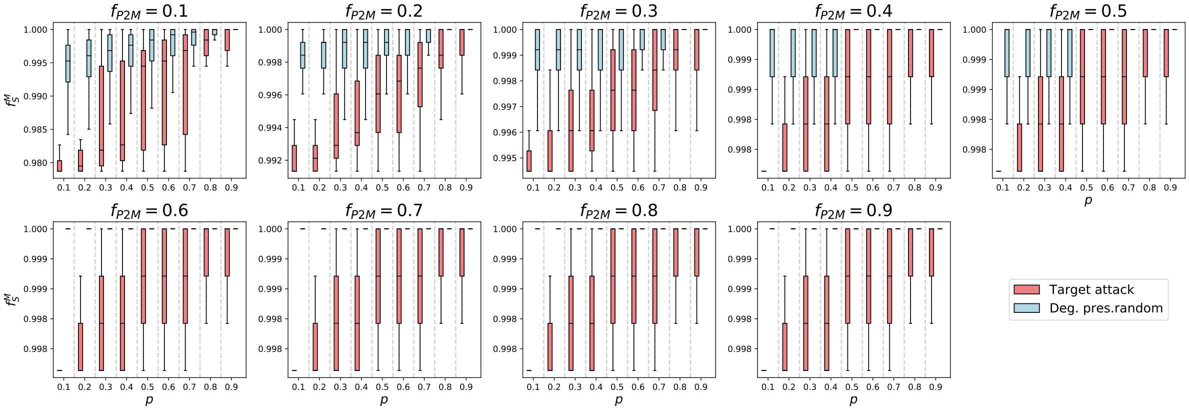

FIG. S13. Targeting blood pressure-related genes (red boxes) causes more damage to the metabolic network than degree-preserving random attacks (blue boxes). Fraction of functional nodes after the perturbations for several values of remaining fraction p of metabolic disease genes as the threshold proportion f_{P2M} varies from 0.1 to 1. Lower values indicate a higher degree of damage to the network. The results are calculated based on 1000 random realizations. For each distribution, boxes indicate the quartiles, whiskers extend to an additional 1.5 * IQR interval, and the medians are represented by a black line.

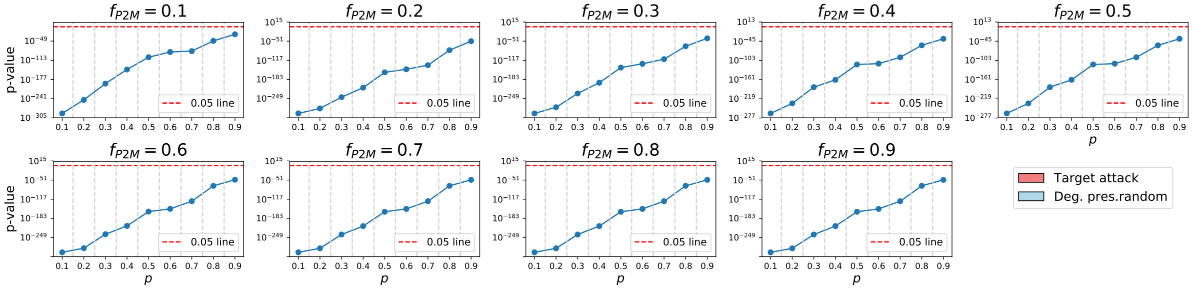

FIG. S14. Statistical analysis of the difference between the targeted attacks on blood pressure-related genes and degree-preserving random attacks. The curves represent the p-values of the one-sided Mann-Whitney test when comparing the two distributions of final functional network size for every value of p and f_{P2M}.

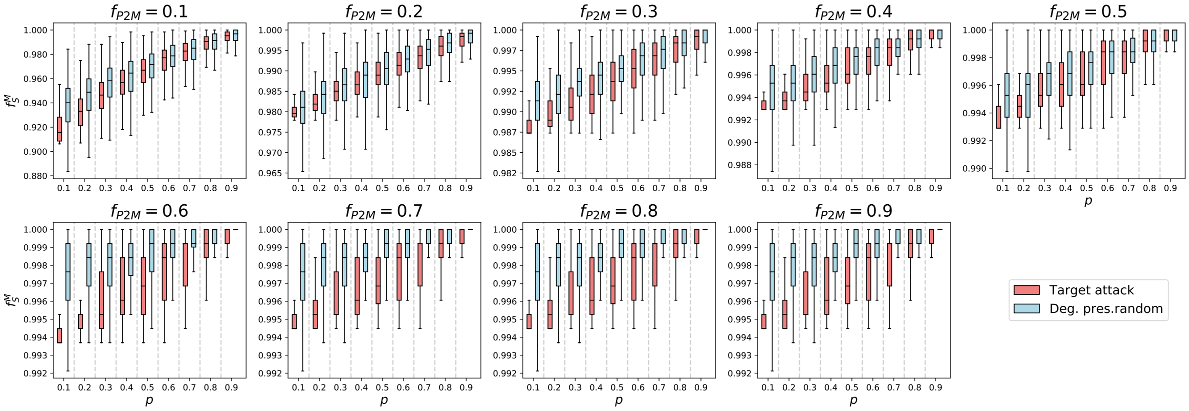

FIG. S15. Targeting t2d-related genes (red boxes) causes more damage to the metabolic network than degree-preserving random attacks (blue boxes). Fraction of functional nodes after the perturbations for several values of remaining fraction p of metabolic disease genes as the threshold proportion f_{P2M} varies from 0.1 to 1. Lower values indicate a higher degree of damage to the network. The results are calculated based on 1000 random realizations. For each distribution, boxes indicate the quartiles, whiskers extend to an additional 1.5 * IQR interval, and the medians are represented by a black line.

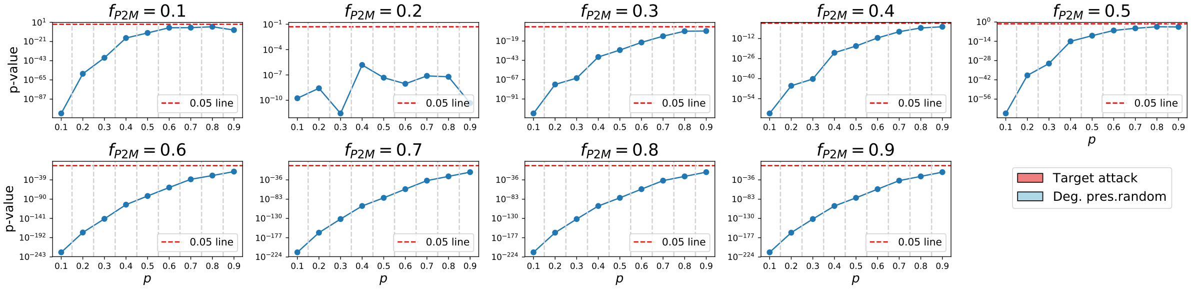

FIG. S16. Statistical analysis of the difference between the targeted attacks on t2d-related genes and degree-preserving random attacks. The curves represent the p-values of the one-sided Mann-Whitney test when comparing the two distributions of final functional network size for every value of p and f_{P2M}.

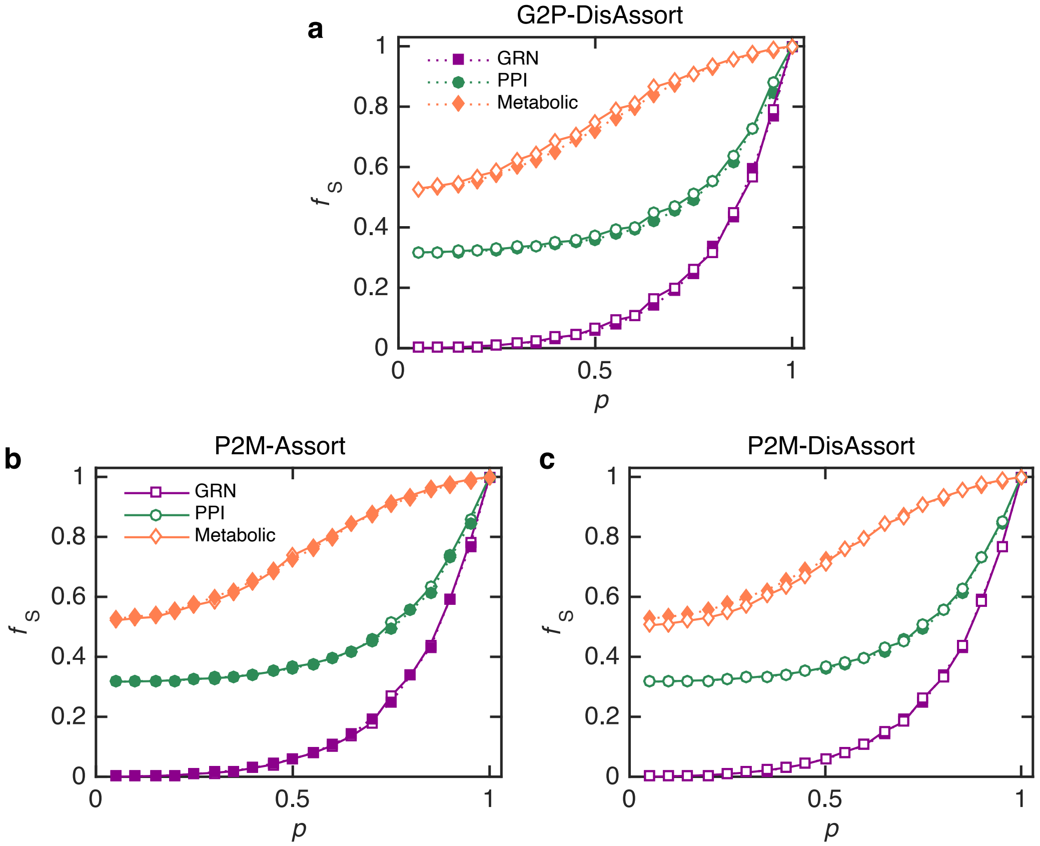

FIG. S17. The real network model has comparable robustness with the disassortatively randomized models. The randomized model in (**a**) is generated by maintenance of the degree distributions in gene regulatory and PPI networks but decreasing the degree correlations between the connected gene-protein pairs. The randomized models in (**b**) and (**c**) are, respectively, generated by maintaining the degree distributions in PPI and metabolic networks, but increasing or decreasing the degree correlations between the connected gene-protein pairs. The results are averaged over 30 realizations.

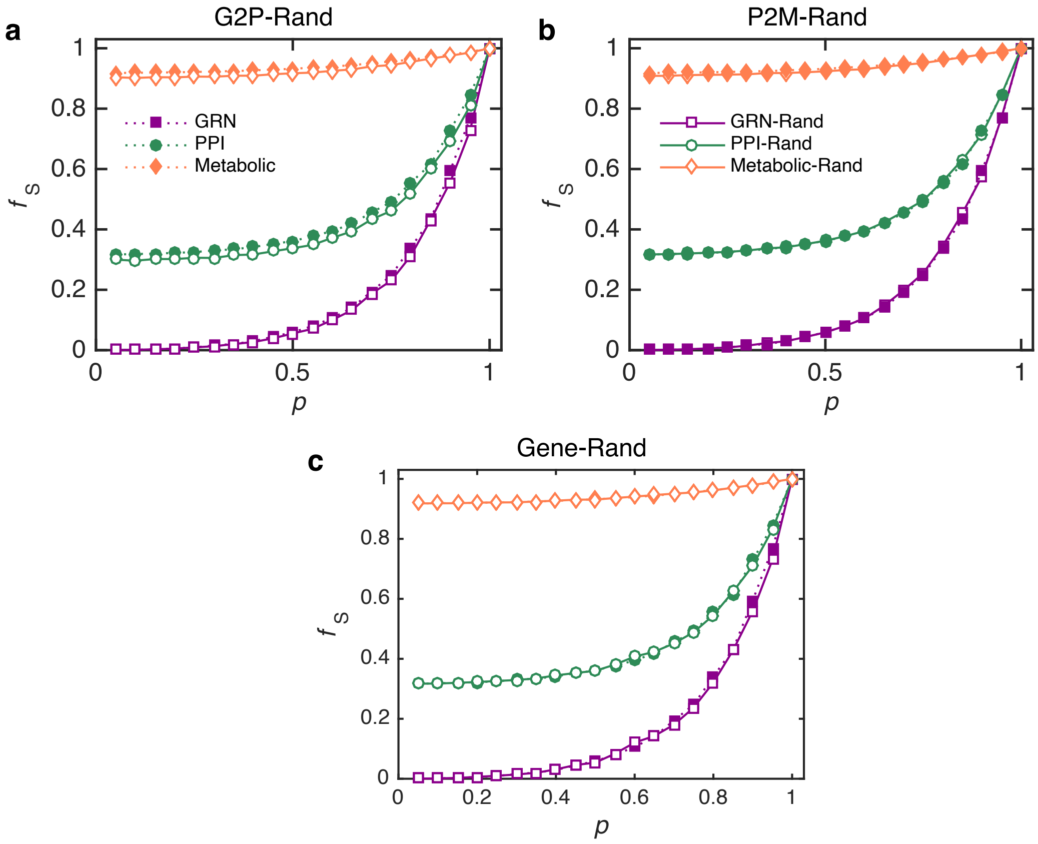

FIG. S18. The real network model has comparable robustness with the neutrally randomized models. The randomized models in (**a**) are obtained by rewiring the gene-protein relations but maintaining the coupling degree distributions in gene regulatory and PPI networks. The randomized models in (**b**) are obtained by maintaining the degree distributions in PPI and metabolic networks and rewiring the protein-metabolites connections. The randomized models in (**c**) are obtained by maintaining the degree distribution of the gene regulatory network and rewiring the links in this layer. The results are averaged over 30 realizations.

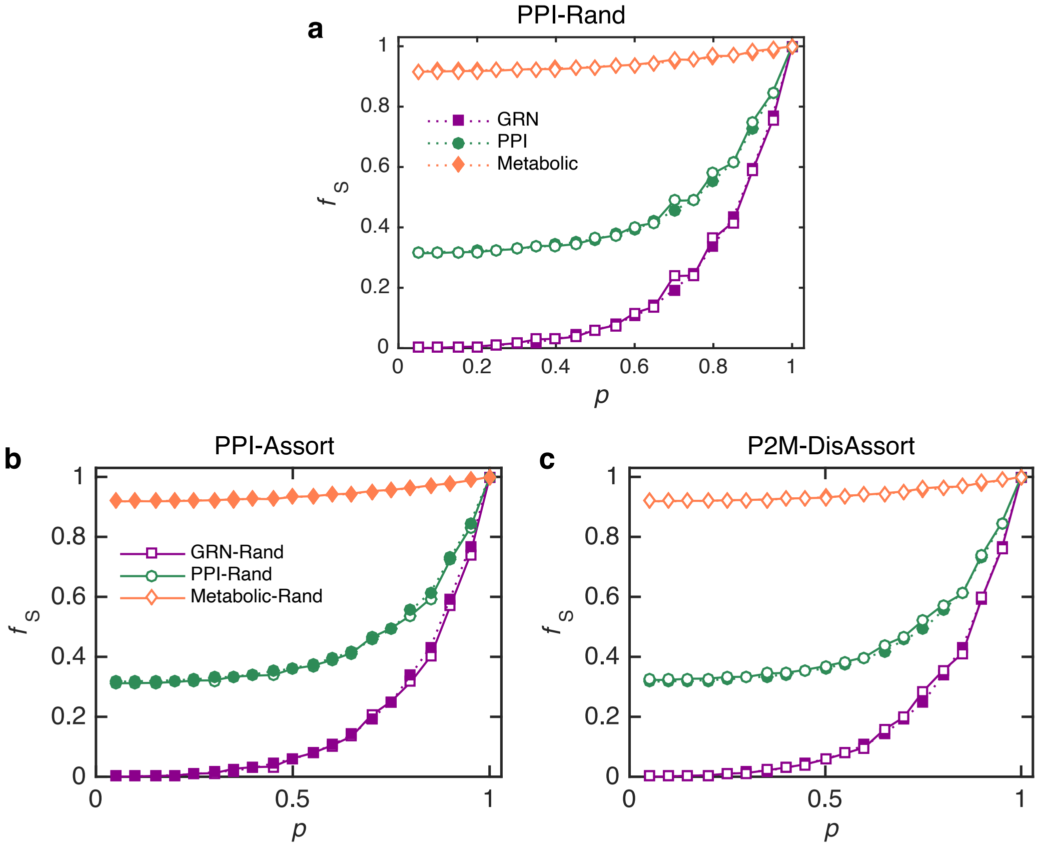

FIG. S19. The real network model has comparable robustness with (**a**) neutrally, (**b**) assortatively, and (**c**) disassortatively randomizations in the PPI network. The randomized models are obtained by rewiring links in PPI network in three different directions, but maintaining the degree distribution. The results are averaged over 30 realizations.

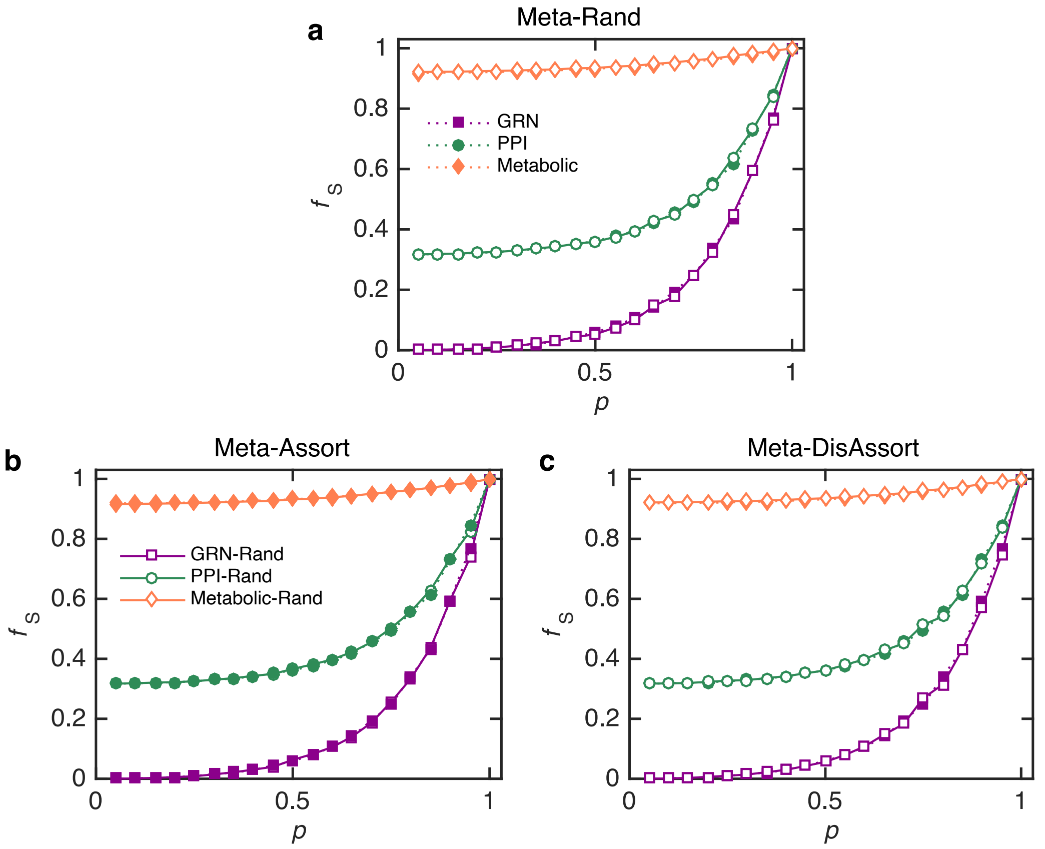

FIG. S20. The real network model has comparable robustness with the (**a**) neutrally, (**b**) assortatively, and (**c**) disassortatively randomizations in the metabolic network. The randomized models are obtained by rewiring links in metabolic network but maintaining the degree distribution. The results are averaged over 30 realizations.

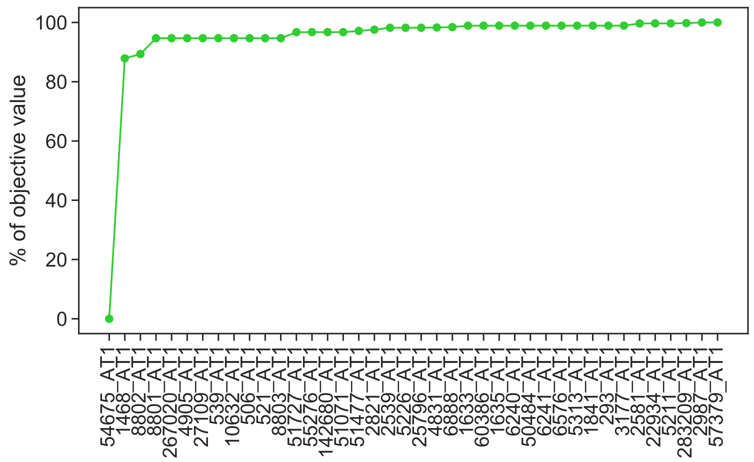

FIG. S21. The percentage of objective value (i.e. growth rate) of the single gene knockout with respect to the wild type objective value (y-axis), plotted for each gene knockout (x-axis), ordered by its impact on the objective value. Only the knockouts that reduce the growth rate by any amount are shown in this figure.

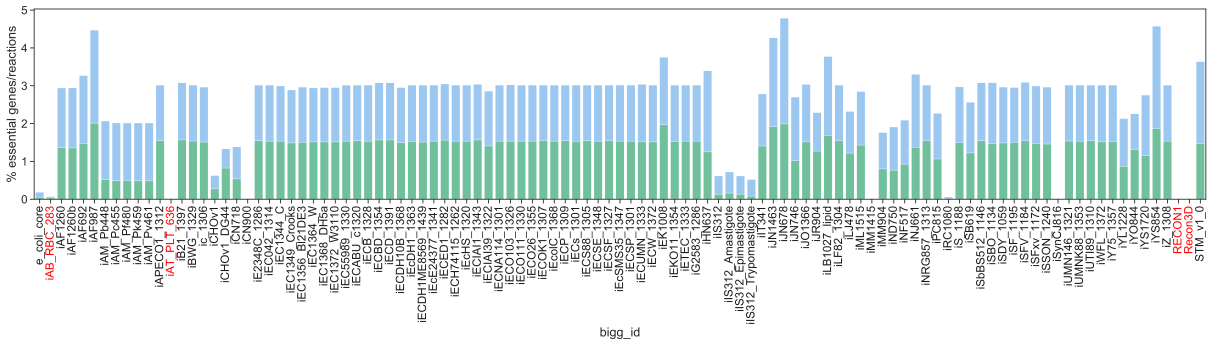

FIG. S22. The percentage of essential genes (green bars) and essential reactions (blue bars) with respect to the total number of genes and reactions (y-axis) in all genome-scale metabolic models in the BiGG database (x-axis). Each bar corresponds to a metabolic model. Human metabolic models are marked in red.

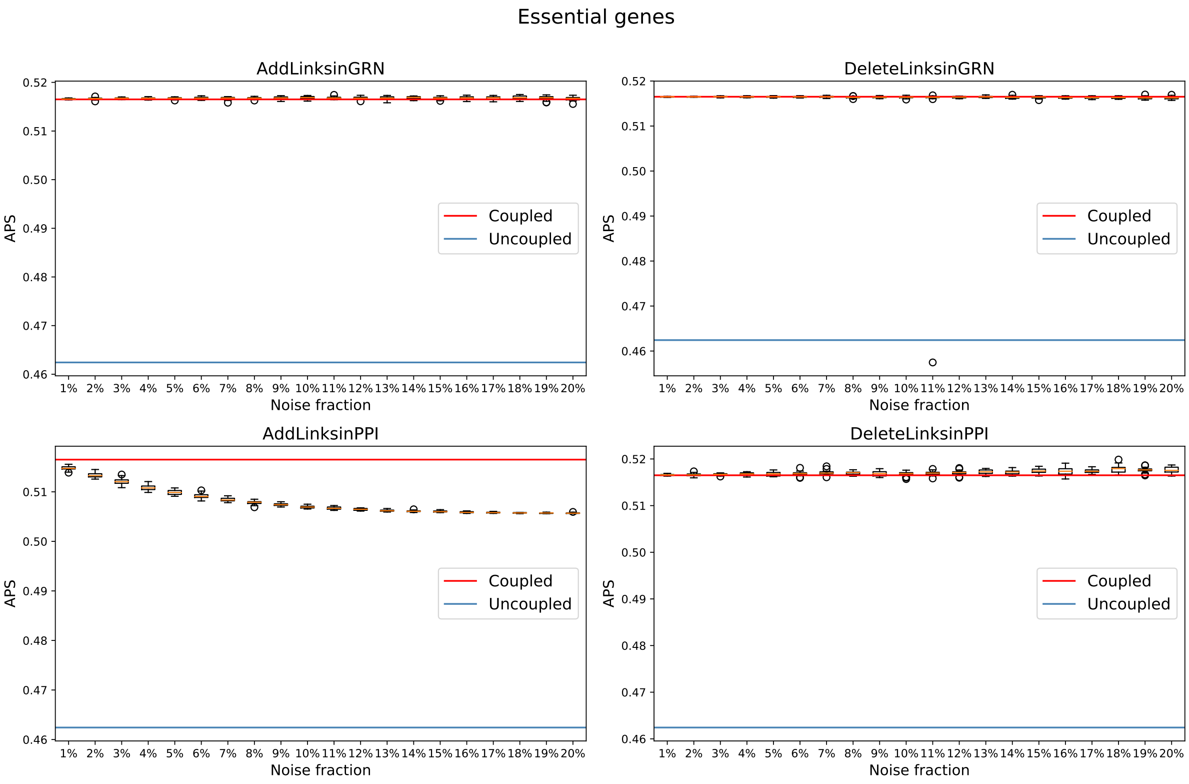

FIG. S23. Distribution of APS values of the prioritization of essential genes in the coupled case with varying amounts of edge noise (1% to 20%) in the case of adding links to the GRN (top left), removing links from the GRN (top right), adding links to the PPI (bottom left), and removing links from the PPI (bottom right). The red and blue lines represent the APS values of the baseline coupled and uncoupled perturbations, respectively. Each distribution is calculated based on 30 random realizations. For each distribution, boxes indicate the quartiles, whiskers extend to an additional 1.5 * IQR interval, and medians are represented by a black line.

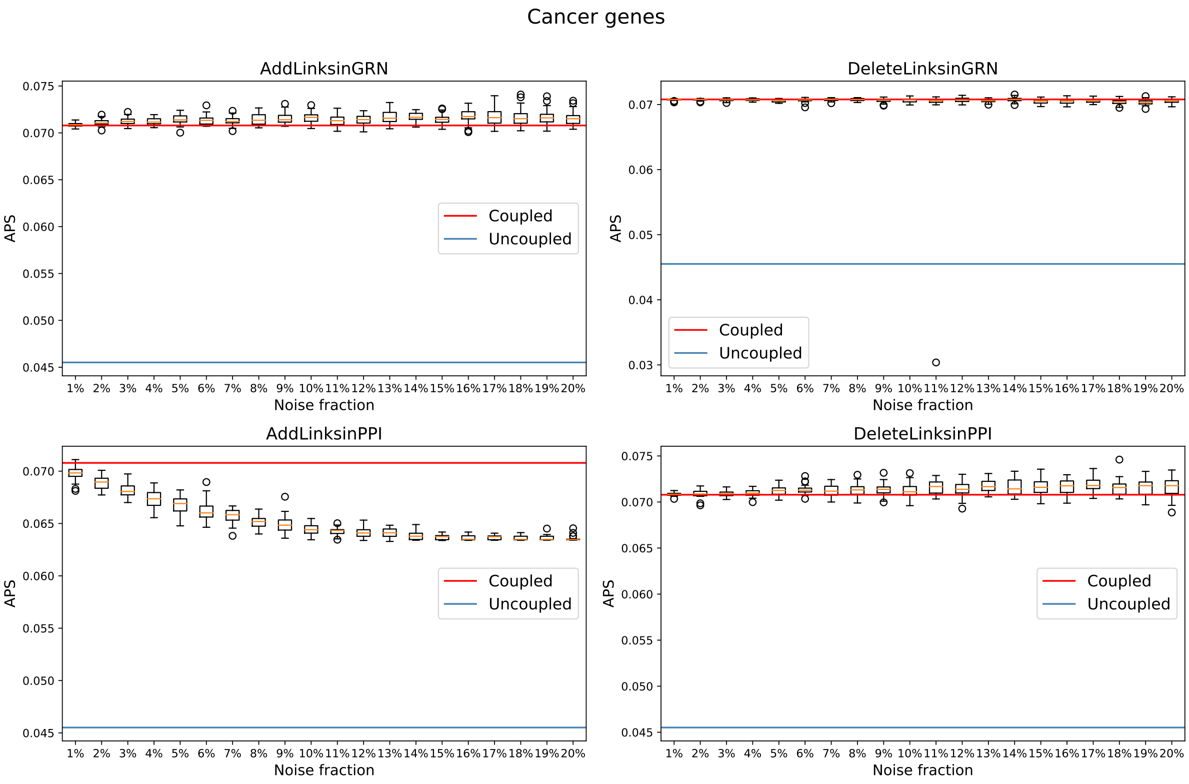

FIG. S24. Distribution of APS values of the prioritization of cancer genes in the coupled case with varying amounts of edge noise (1% to 20%) in the case of adding links to the GRN (top left), removing links from the GRN (top right), adding links to the PPI (bottom left), and removing links from the PPI (bottom right). The red and blue lines represent the APS values of the baseline coupled and uncoupled perturbations, respectively. Each distribution is calculated based on 30 random realizations. For each distribution, boxes indicate the quartiles, whiskers extend to an additional 1.5 * IQR interval, and medians are represented by a black line.

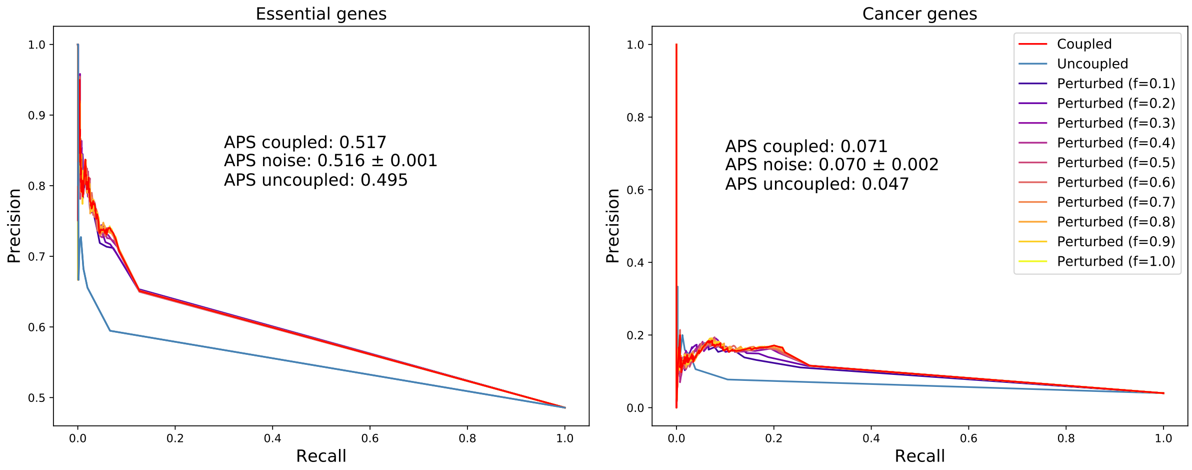

FIG. S25. Precision-recall curves of the prioritization of essential (left) and cancer (right) genes in the coupled case (red) as compared to the coupled case with varying values of f (0.1 to 1, multiple colors) and uncoupled case (blue). Note that f=1 corresponds to the baseline coupled case.

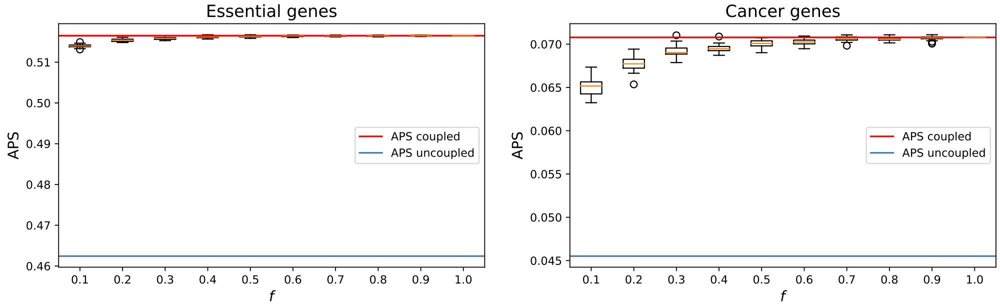

FIG. S26. Distribution of average precision scores (APS) of the prioritization of essential (left) and cancer (right) genes in the coupled case (red) over different realizations of the process for each value of f. Each distribution is calculated based on 30 random realizations. For each distribution, boxes indicate the quartiles, whiskers extend to an additional 1.5 * IQR interval, and medians are represented by a black line.

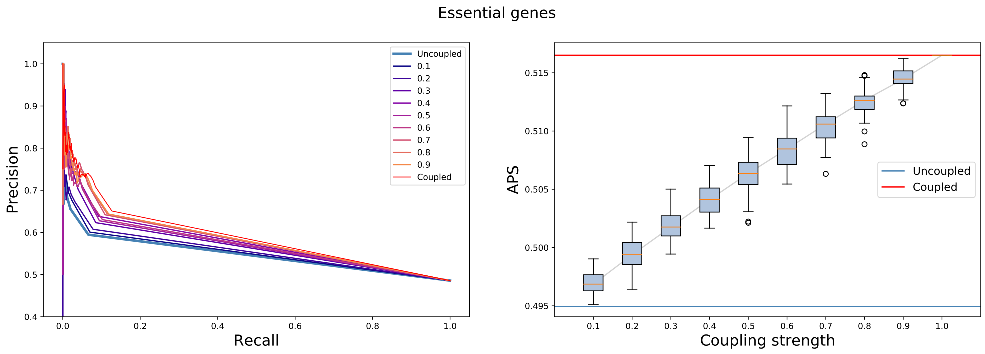

FIG. S27. Precision-recall curves (left) and distribution of APS values (right) of the prioritization of essential genes in the coupled case (red), uncoupled case (blue) and coupled case in the case of varying degree of coupling strengths between the GRN and PPI network. Each distribution is calculated based on 30 random realizations. For each distribution, boxes indicate the quartiles, whiskers extend to an additional 1.5 * IQR interval, and medians are represented by a black line.

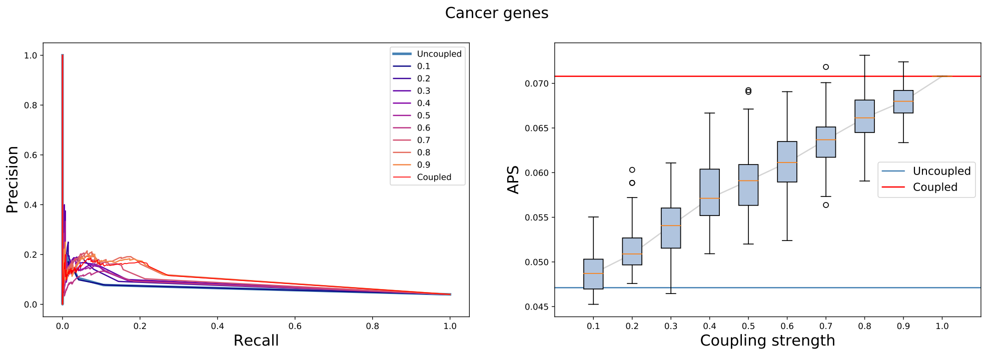

FIG. S28. Precision-recall curves (left) and distribution of APS values (right) of the prioritization of cancer genes in the coupled case (red), uncoupled case (blue) and coupled case in the case of varying degree of coupling strengths between the GRN and PPI network. Each distribution is calculated based on 30 random realizations. For each distribution, boxes indicate the quartiles, whiskers extend to an additional 1.5 * IQR interval, and medians are represented by a black line.

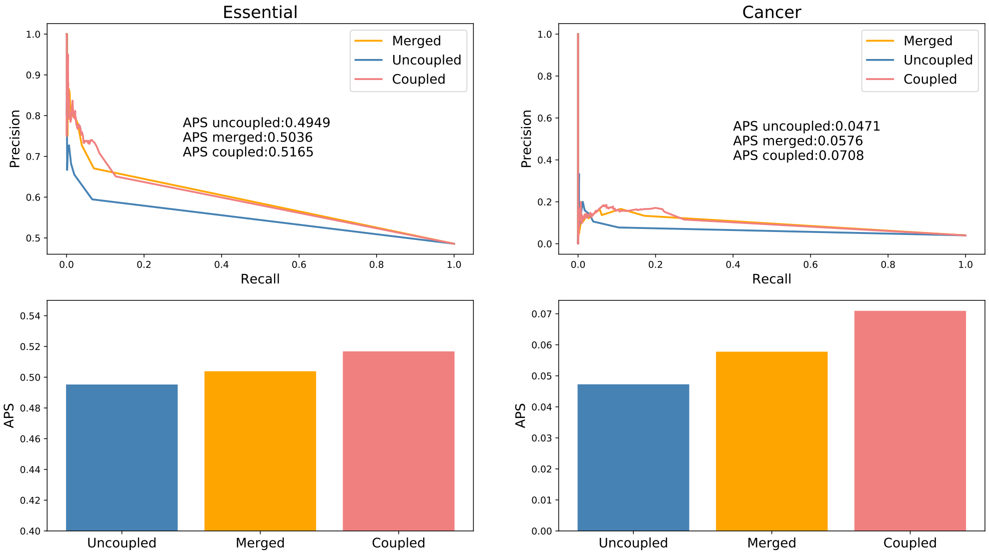

FIG. S29. Precision-recall curves (top row) and APS values (bottom row) of the prioritization of essential (left column) and cancer (right column) genes in the coupled case (red), uncoupled case (blue) and uncoupled case in the merged network (orange)

[1] D. F. Klosik, A. Grimbs, S. Bornholdt, and M.-T. Hütt, Nature communications **8**, 534 (2017).

[2] I. Bartha, J. di Iulio, J. C. Venter, and A. Telenti, Nature Reviews Genetics **19**, 51 (2018).

[3] M. E. Dickinson, A. M. Flenniken, X. Ji, L. Teboul, M. D. Wong, J. K. White, T. F. Meehan, W. J. Weninger, H. Westerberg, H. Adissu, *et al.*, Nature **537**, 508 (2016).

[4] A. Ebrahim, J. A. Lerman, B. O. Palsson, and D. R. Hyduke, BMC systems biology **7**, 74 (2013).

[5] J. G. Cardoso, K. Jensen, C. Lieven, A. S. Lærke Hansen, S. Galkina, M. Beber, E. Ozdemir, M. J. Herrgård, H. Redestig, and N. Sonnenschein, ACS synthetic biology **7**, 1163 (2018).

[6] J. Edwards and B. Palsson, Proceedings of the National Academy of Sciences **97**, 5528 (2000).

[7] F. Gatto, H. Miess, A. Schulze, and J. Nielsen, Scientific reports **5**, 10738 (2015).

[8] E. Brunk, S. Sahoo, D. C. Zielinski, A. Altunkaya, A. Dräger, N. Mih, F. Gatto, A. Nilsson, G. A. P. Gonzalez, M. K. Aurich, *et al.*, Nature biotechnology **36**, 272 (2018).
