# Supplementary material for: Robustness and lethality in multilayer biological molecular networks
Source: Nat Commun. 2020 Nov 27;11:6043. doi: 10.1038/s41467-020-19841-3 (PMC7699651; doi:10.1038/s41467-020-19841-3)
Supplement: Supplementary file 2 — Reporting Summary [file 41467_2020_19841_MOESM2_ESM.pdf]

nature research

Corresponding author(s): Jianxi Gao, Amitabh Sharma

Last updated by author(s): Oct 12, 2020

Reporting Summary

Nature Research wishes to improve the reproducibility of the work that we publish. This form provides structure for consistency and transparency in reporting. For further information on Nature Research policies, see our Editorial Policies and the Editorial Policy Checklist.

Statistics

For all statistical analyses, confirm that the following items are present in the figure legend, table legend, main text, or Methods section.

n/a	Confirmed	
☒	☐	The exact sample size (*n*) for each experimental group/condition, given as a discrete number and unit of measurement
☒	☐	A statement on whether measurements were taken from distinct samples or whether the same sample was measured repeatedly
☐	☒	The statistical test(s) used AND whether they are one- or two-sided<br>*Only common tests should be described solely by name; describe more complex techniques in the Methods section.*
☐	☒	A description of all covariates tested
☒	☐	A description of any assumptions or corrections, such as tests of normality and adjustment for multiple comparisons
☒	☐	A full description of the statistical parameters including central tendency (e.g. means) or other basic estimates (e.g. regression coefficient) AND variation (e.g. standard deviation) or associated estimates of uncertainty (e.g. confidence intervals)
☐	☒	For null hypothesis testing, the test statistic (e.g. *F*, *t*, *r*) with confidence intervals, effect sizes, degrees of freedom and *P* value noted<br>*Give P values as exact values whenever suitable.*
☒	☐	For Bayesian analysis, information on the choice of priors and Markov chain Monte Carlo settings
☒	☐	For hierarchical and complex designs, identification of the appropriate level for tests and full reporting of outcomes
☒	☐	Estimates of effect sizes (e.g. Cohen's *d*, Pearson's *r*), indicating how they were calculated

Our web collection on statistics for biologists contains articles on many of the points above.

Software and code

Policy information about availability of computer code

Data collection	No software was used.
Data analysis	The main results are generated by using Matlab-R2017a, Python2.7 and R-3.6.1. The code generating the main results in this work are available at https://github.com/XuemingLiu/Robust-Multilyer-BioNetworks.

For manuscripts utilizing custom algorithms or software that are central to the research but not yet described in published literature, software must be made available to editors and reviewers. We strongly encourage code deposition in a community repository (e.g. GitHub). See the Nature Research guidelines for submitting code & software for further information.

Data

Policy information about availability of data

All manuscripts must include a data availability statement. This statement should provide the following information, where applicable:

- Accession codes, unique identifiers, or web links for publicly available datasets
- A list of figures that have associated raw data
- A description of any restrictions on data availability

The data used in this work are collected from open-access databases.
The transcription factors and their DNA binding motifs used in the construction of the human regulatory network were downloaded from the CIS-BP database (http://cisbp.ccbr.utoronto.ca/). The three tissue-specific gene regulatory networks were downloaded from the FANTOM5 database [59], respectively “forebrain” (ID: 03), “lymphocytes” (ID: 12), and “lung” (ID: 23). The PPI network data were obtained from Supplementary Data 1 provided by Cheng et al. [60]. The human chemical-chemical (chemical_chemical.links.detailed.v4.0.tsv) and protein-biochemical (9606.protein_chemical.links.detailed.v4.0.tsv) links were downloaded from the STITCH database (version 4.0) (http://stitch.embl.de/). The human metabolites data were downloaded from the Human Metabolome Database (HMDB) (version 3.5, retrieved November 2015). The essential and essential genes were respectively collected from the Database of Essential Genes

(DEG) [68] and the Cancer Gene Census (CGC) [70]. The metabolic disease associated genes were integrated from large scale genome-wide association studies (GWAS) published in the literature and data from the NHGRI-EBI GWAS Catalog (https://www.ebi.ac.uk/gwas/) [80]. The authors declare that the data supporting the findings of this study are available from the corresponding author upon request.

Field-specific reporting

Please select the one below that is the best fit for your research. If you are not sure, read the appropriate sections before making your selection.

- [x] Life sciences
- [ ] Behavioural & social sciences
- [ ] Ecological, evolutionary & environmental sciences

For a reference copy of the document with all sections, see nature.com/documents/nr-reporting-summary-flat.pdf

Life sciences study design

All studies must disclose on these points even when the disclosure is negative.

Sample size	The statistical testing performed in this paper is based on samples populations generated by numerical randomization, and therefore sample size calculation is not applicable for this work
Data exclusions	No data were excluded from the analysis
Replication	For the reasons stated above standard replication procedures were not performed. However, we validated several of our findings on alternative datasets. For example, we validated the results on the essential gene enrichment on three additional criteria for gene essentiality proposed in literature. As another example, we validated the finding on the network robustness on three additional tissue specific regulatory networks built as described in Methods.
Randomization	Since this is not a case/control study and for the reasons stated above, randomization is not relevant for this study
Blinding	Since this is not a case/control study and for the reasons stated above, blinding is not relevant for this study

Reporting for specific materials, systems and methods

We require information from authors about some types of materials, experimental systems and methods used in many studies. Here, indicate whether each material, system or method listed is relevant to your study. If you are not sure if a list item applies to your research, read the appropriate section before selecting a response.

Materials & experimental systems

n/a	Involved in the study	
☒	☐	Antibodies
☒	☐	Eukaryotic cell lines
☒	☐	Palaeontology and archaeology
☒	☐	Animals and other organisms
☒	☐	Human research participants
☒	☐	Clinical data
☒	☐	Dual use research of concern

Methods

n/a	Involved in the study	
☒	☐	ChIP-seq
☒	☐	Flow cytometry
☒	☐	MRI-based neuroimaging
